# Spectroscopy of cluster aerosol models: IR and UV spectra of hydrated glyoxylate with and without sea salt†

**DOI:** 10.1039/d3ea00039g

**Published:** 2023-08-30

**Authors:** Nina K. Bersenkowitsch, Sarah J. Madlener, Jakob Heller, Christian van der Linde, Milan Ončák, Martin K. Beyer

**Affiliations:** a Institut für Ionenphysik und Angewandte Physik, Universität Innsbruck Technikerstraße 25 6020 Innsbruck Austria milan.oncak@uibk.ac.at martin.beyer@uibk.ac.at

## Abstract

Glyoxylic acid is formed in the troposphere by oxidation of organic molecules. In sea salt aerosols, it is expected to be present as glyoxylate, integrated into the salt environment and strongly interacting with water molecules. In water, glyoxylate is in equilibrium with its *gem*-diol form. To understand the influence of water and salt on the photophysics and photochemistry of glyoxylate, we generate small model clusters containing glyoxylate by electrospray ionization and study them by Fourier-Transform Ion Cyclotron Resonance (FT-ICR) mass spectrometry. We used infrared multiple photon dissociation spectroscopy and UV/vis photodissociation spectroscopy for structural characterization as well as quantum chemical calculations to model the spectra and dissociation patterns. Resonant absorption of infrared radiation leads to water evaporation, which indicates that water and glyoxylate are separate molecular entities in a significant fraction of the clusters, in line with the observed absorption of UV light in the actinic region. Hydration of glyoxylate leads to a change of the dihedral angle in the CHOCOO^−^·H_2_O complex, causing a slight redshift of the S_1_ ← S_0_ transition. However, the barriers for internal rotation are below 5 kJ mol^−1^, which explains the broad S_1_ ← S_0_ absorption extending from about 320 to 380 nm. Most importantly, hydration hinders dissociation in the S_1_ state, thus enhancing the quantum yield of fluorescence combined with water evaporation. No C–C bond photolysis is observed, but due to the limited signal-to-noise ratio, it cannot be ruled out. The quantum yield, however, will be relatively low. Fluorescence dominates the photophysics of glyoxylate embedded in the dry salt cluster, but the quantum yield shifts towards internal conversion upon addition of one or two water molecules.

## Abstract 2

Tropospheric glyoxylic acid is most likely converted to glyoxylate in sea salt aerosols. We report on the influence of water and salt ions on the structure and photochemistry of glyoxylate.

## Graphical abstract

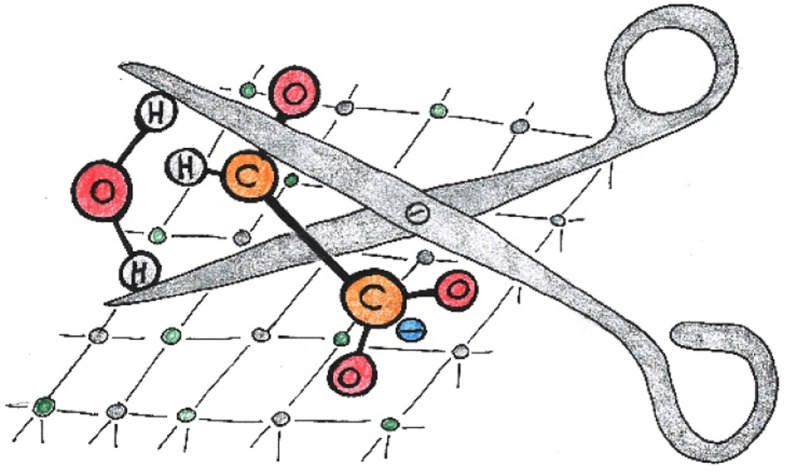

**Environmental significance**

Many organic trace compounds in the troposphere are processed in sea salt aerosols, by a complex interplay of photochemistry and ground-state chemical reactions. A detailed understanding of the elementary steps involved is still lacking. Here, we use glyoxylate, water, and sodium chloride to build laboratory models. We elucidate the structure of glyoxylate in different model systems and study the influence of water and salt on its photochemistry. We learn that the dihedral angle between the carboxylate and the formyl group is sensitive to changes in the immediate environment, and at the same time crucial for the energetics of photochemically relevant excited states. Photochemical reactions and relaxation pathways are also modified by the interaction with water and salt ions.

## Introduction

The climate on Earth is a highly complex system that is influenced by many physical and chemical processes. In order to predict the development of the climate, accurate models that include all relevant effects are required. Especially for long-term projections, the inclusion of aerosol^1^ effects is essential. Aerosols influence the climate both directly^2^ and indirectly^3^ as they can absorb or backscatter^4^ sunlight and provide condensation nuclei for clouds.^5–7^ Solar radiation plays a pivotal role in reactions on aerosols^8^ and might initiate various chemical reactions^9,10^ or photoisomerization.^11,12^ As the ocean covers more than 70% of the Earth's surface, marine aerosols^13^ appear with quite high concentrations in these regions and contribute significantly to the regional but also global climate. The origin of these aerosols is traced back to sea spray,^14,15^*i.e.* tiny droplets emitted into the troposphere *via* breaking of waves or mechanical turning of the water surface by wind. Beside water and sea salt, marine aerosols are highly complex systems containing a vast variety of organic^6,16–19^ as well as inorganic species.^19^

Organic acids^20–22^ were unambiguously identified by several studies as a significant group in aerosols. Glyoxylic acid is the simplest 2-oxocarboxylic acid. It was found to be a secondary oxidation product from isoprene,^23,24^ (methyl-)glyoxal^25,26^ and aromatic hydrocarbons,^27^ being routinely identified in outdoor samples.^28–33^ Glyoxylic acid has three strong absorption bands. One of them lies at around 230 nm, and a highly structured spectrum is found for the actinic region *λ* > 290 nm, *i.e.* the wavelength range of solar radiation that reaches the troposphere. The major products of the photolysis of neutral glyoxylic acid are CO_2_ and formaldehyde, while small amounts of H_2_ and CO are found as well.^34^ Collisional activation of glyoxylate leads to decarboxylation forming HCO^−^,^35^ as found by Miller and Uggerud. While no spectroscopic work on bare or hydrated glyoxylate is available in the gas phase, the groups of Verlet^36^ and Wang^37^ have recently studied the closely related pyruvate and hydrated pyruvate ions by photoelectron spectroscopy. They found efficient photolysis of bare pyruvate, with hydration reducing the quantum yield of the photodissociation channels.

We have recently studied a series of salt clusters containing organic molecules by both infrared spectroscopy^38–43^ and UV/vis spectroscopy,^43–45^ always in combination with high-level quantum chemistry. We were able to show that the photochemistry of glyoxylate in dry sea salt clusters differs from gaseous glyoxylate, resembling more closely the photochemistry of glyoxylic acid.^45^ Here, we take it a step further and include water molecules into the cluster, to identify potential changes in the spectra and photochemical reactions upon hydration. Specifically, if the aldehyde group of glyoxylate can rearrange to its geminal diol form, short *gem*-diol, in the presence of water. Such a transformation would change the photochemistry and therefore the spectra drastically, as the *gem*-diol structure does not absorb in the actinic region. The *gem*-diol form of glyoxylic acid was studied theoretically^46^ and experimentally in aqueous solution^47^ as well as in the gas phase.^48^

In this work, we start with infrared multiple photon dissociation (IRMPD) of cationic sodium chloride clusters doped with one glyoxylate ion and one water molecule to test whether the *gem*-diol form plays a major role in such gas-phase systems. The influence of water on the photochemistry of glyoxylate is investigated by UV/vis photodissociation of the glyoxylate–water complex. Water-doped sodium-glyoxylate clusters Na_3_(C_2_HO_3_)_2_(H_2_O)_*m*_^+^, *m* = 0–2, are then used to probe the combined effect of sodium ions and water. Quantum chemical calculations are used to interpret the experimental findings and to deepen the understanding of the mechanisms involved.

## Experimental and theoretical methods

The experimental setup and procedure has been described in detail elsewhere.^49^ Ions are produced by electrospray ionization (ESI) and guided *via* a sequence of two funnels, a quadrupole mass filter, a hexapole collision cell and an electrostatic lens system into the cell of a Bruker Apex Qe 9.4 T Fourier-Transform Ion Cyclotron Resonance Mass Spectrometer (FT-ICR MS), where they are stored and mass selected. A solution of 5 mmol l^−1^ Na^35^Cl (99% ^35^Cl, Sigma-Aldrich) and 1 mmol l^−1^ glyoxylate monohydrate (98%, Sigma-Aldrich) in water (HPLC grade, Sigma-Aldrich) was used for ESI. In the experiments measuring the photodissociation spectrum of [C_2_HO_3_·H_2_O]^−^, traces of ethanol were added to improve the ion yield. Optical parametric oscillator/amplifier (OPO/OPA) systems are coupled into the ICR cell to enable the performance of action spectroscopy. Both UV photodissociation spectra (EKSPLA NT342B, 225–2600 nm) and infrared multiple photon dissociation (IRMPD) spectra (EKSPLA NT277, 2600–4475 nm) were recorded. The laser light is introduced from the back of the instrument through a window into the ICR cell. Irradiation time is adapted for the different regions of the spectrum so that also very weak photodissociation cross sections can be quantified, which are expected in particular for the actinic region of the UV/vis spectra. The intensities of all fragments *I*_*i*_ relative to the original ion *I*_0_, laser pulse energy *E* and laser beam diameter *A*, as well as the number of pulses *p*, the wavelength *λ* and the irradiation time *t*_irr_ are used for calculating photodissociation cross sections, eqn (1). Also, the influence of black body infrared dissociation (BIRD) has been taken into account. The error for the absolute cross sections, which are presented in this work, is estimated to be around 30%. Reasons for this error are mostly the measured BIRD rate constant, *k*_BIRD_, and the photon flux in the ICR cell.^45^ For IR cross sections, multiphoton analysis was performed as described in detail earlier.^38^1
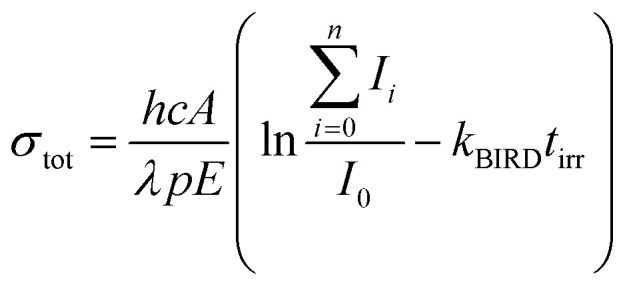


A series of isomers of the investigated clusters in the electronic ground state were calculated using density functional theory (DFT) on the B3LYP/def2TZVP level. To obtain more reliable energies, single-point calculations on the optimized structures were performed using Møller–Plesset perturbation theory (MP2), employing the aug-cc-pVDZ basis set. Small clusters were also optimized using coupled cluster singles and doubles (CCSD) method. For sodium chloride clusters doped with one glyoxylate and one water molecule, structures of glyoxylate doped clusters from a previous study were taken,^45^ adding H_2_O at different positions. For Na_*n*_Cl_*n*−2_(C_2_HO_3_)(H_2_O)^+^, *n* = 4, 5, we started optimization from 51 and 17 isomers, respectively. For *n* = 6–11, at least 13 initial structures were considered for optimization. For Na_3_(C_2_HO_3_)_2_(H_2_O)_*m*_^+^, *m* = 0–2, at least four isomers were considered as starting points for optimization. Calculated IR frequencies were scaled with an empirical factor of 0.96. All reaction energies are zero-point corrected but with no further thermal correction.

Excited state calculations were performed using the Equation of Motion CCSD (EOM-CCSD), time-dependent DFT (TD-DFT) with the CAM-B3LYP functional (employing natural transition orbitals, NTO, analysis^50^ to obtain the excitation character), and multi-reference methods, namely complete active space self-consistent field (CASSCF) and multi-reference configuration interaction (MRCI). To model the absorption spectrum of the [C_2_HO_3_·H_2_O]^−^ system, ground state sampling was performed for the two most stable isomers found as well as the *gem*-diol anion using molecular dynamics with a time step of 30 a.u. (∼0.73 fs) at a temperature of 300 K maintained by a Nosé–Hoover chain thermostat. After a thermalization period of 5000 steps, 200 structures per isomer were taken every 500 steps for spectrum modeling. The absorption spectra were modeled using the reflection principle.^51–53^ When processing the spectrum, the calculated EOM-CCSD/aug-cc-pVDZ transitions were convoluted by Gaussian functions with full width at half maximum (FWHM) of 0.15 eV. UV spectra of larger Na_3_(C_2_HO_3_)_2_(H_2_O)_*n*_^+^, *n* = 0–2, clusters were modeled at the TD-CAM-B3LYP/aug-cc-pVDZ//B3LYP/def2TZVP level within the linearized reflection principle formalism.^53^ Quantum chemical calculations were performed in the Gaussian package,^54^ multi-reference calculations within the Molpro program,^55,56^ molecular dynamics calculations in the Abin program.^57,58^

## Results and discussion

### Calculations of glyoxylate structure in different environments

**Fig. 1:**
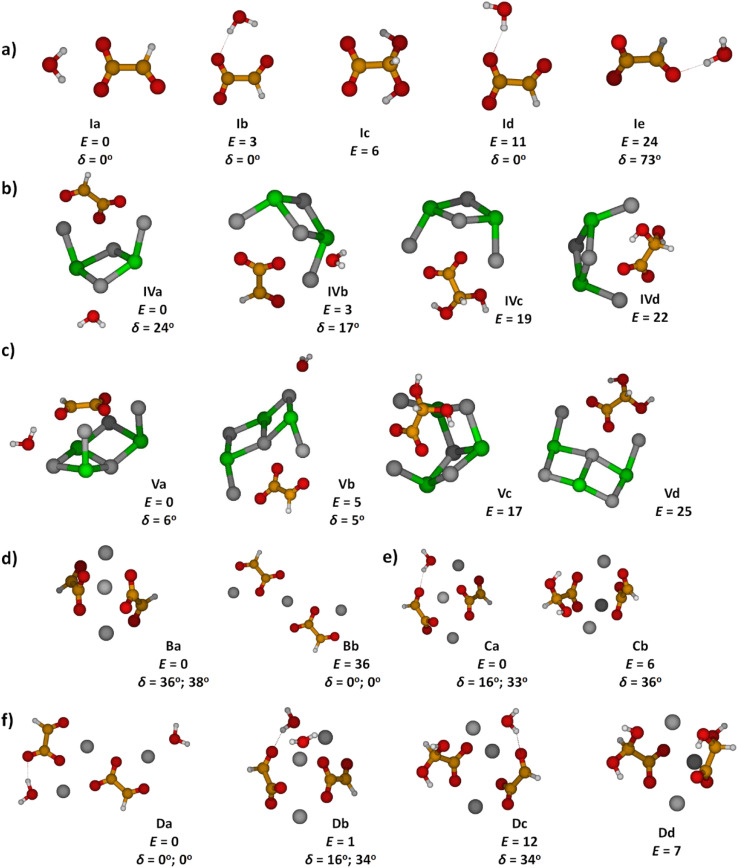
Calculated structures, relative energies *E* in kJ mol^−1^ and OCCO dihedral angle *δ* in glyoxylate for (a) glyoxylate–water complexes; (b and c) two hydrated salt-glyoxylate clusters, either as Na_*n*_Cl_*n*−2_(C_2_HO_3_)(H_2_O)^+^ or in its *gem*-diol form, Na_*n*_Cl_*n*−2_(COOC(OH)_2_)^+^, for *n* = 4 and *n* = 5, a, b isomers include C_2_HO_3_^−^·H_2_O, others the *gem*-diol form; see Fig. S1† for further *n* = 4 isomers and Fig. S2† for *n* = 6–11; (d–f) isomers of Na_3_(C_2_HO_3_)_2_(H_2_O)_*m*_^+^, *m* = 0–2, stoichiometry, order with the increasing number of *gem*-diol moieties. Calculated at CCSD/aug-cc-pVDZ (a) and MP2/aug-cc-pVDZ//B3LYP/def2TZVP (b–f) levels. Color code: Na grey, Cl green, C orange, O red, H white.

Optimized structures of energetically low-lying isomers of the studied systems in the native glyoxylate as well as the *gem*-diol form are summarized in Fig. 1. Isomers of [C_2_HO_3_·H_2_O]^−^ shown in Fig. 1a include the hydrogen-bonded complex as well as the *gem*-diol form, COOCH(OH)_2_^−^. At the CCSD level, the most stable glyoxylate–water isomer is calculated to be 6 kJ mol^−1^ lower than the *gem*-diol form, this value is however close to the computational uncertainty. Interestingly, hydration at the carbonate group changes the dihedral angle *δ*(OCCO) from 64° in glyoxylate to 0° in Ia,b,d, while it stays at 73° in Ie, with the H_2_O hydrogen bonded to the aldehyde O atom. However, the potential is very flat with respect to the OCCO dihedral angle (see below).

In Fig. 1b and c, we show calculated structures of Na_*n*_Cl_*n*−2_(C_2_HO_3_)(H_2_O)^+^ for *n* = 4 and *n* = 5, respectively (see Fig. S1 and S2† for further isomers). When glyoxylate and water are embedded in the salt clusters, the equilibrium seems to be again shifted towards the glyoxylate–water structure due to a less effective interaction of the *gem*-diol with the salt cluster, most probably because of more pronounced disruption of the salt cluster conformation. Water does not necessarily interact directly with the glyoxylate anion in the most stable isomers found, and is also found to prefer the ion–dipole interaction, H_2_O⋯Na^+^. In the presence of salt ions, the OCCO dihedral angle values spread considerably.

In Na_3_(C_2_HO_3_)_2_(H_2_O)_*m*_^+^, *m* = 0–2, the *gem*-diol form is again less stable compared to glyoxylate–water, the difference is however below 15 kJ mol^−1^ (Fig. 1d–f). Note that the structure with two *gem*-diol moieties, Na_3_(COOCH(OH)_2_)_2_, Dd, lies only 7 kJ mol^−1^ above Na_3_(C_2_HO_3_)_2_(H_2_O)_2_^+^, Da. Under thermal conditions, the presence of *gem*-diol isomers cannot be ruled out based on the calculations, but one might expect that the majority of clusters contains glyoxylate in its native form, with a water molecule either hydrogen bonding to glyoxylate or coordinated to a sodium ion.

### IRMPD spectroscopy of sodium chloride clusters doped with glyoxylate and water

**Fig. 2:**
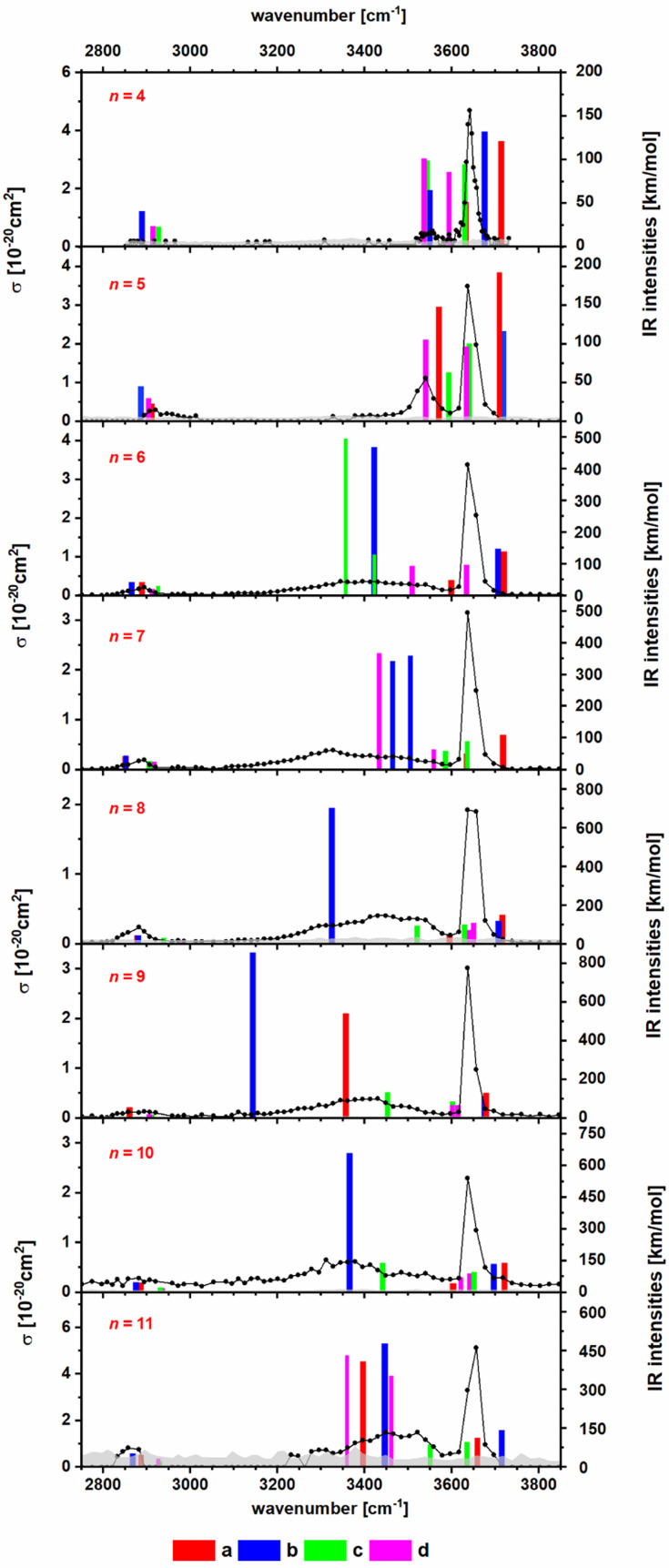
IR photodissociation spectra of the clusters Na_*n*_Cl_*n*−2_(C_2_HO_3_)(H_2_O)^+^ with *n* = 4–11 with an irradiation time of 5 s. Here only one photon cross sections are shown. In Fig. S3,† the plot displaying the two photon cross sections can be found. Theoretical spectra are calculated at the B3LYP/def2TZVP level of theory and are scaled with a factor of 0.96. The isomers a and b are representations for the clusters with an intact glyoxylate and water molecule and isomers c and d represent isomers in the *gem*-diol form. For optimized geometries of isomers a–d, see Fig. 1 and S2.†

To obtain insight into the structure of the Na_*n*_Cl_*n*−2_(C_2_HO_3_)(H_2_O)^+^ clusters, *n* = 4–11, we measured their IRMPD spectra, Fig. 2. The only fragmentation channel observed in the measured wavelength range is water evaporation, requiring 51–75 kJ mol^−1^ according to our calculations, see Tables 1 and S1,† with a strong dependence on cluster size. For *n* = 4, the water binding energy slightly exceeds the thermal energy present in the ion at *T* = 300 K; for larger clusters, the thermal energy is higher than the energy needed to dissociate a water molecule. Thus, one photon might suffice for water loss. For comparison, dissociation of a NaCl unit from the salt cluster requires 180–220 kJ mol^−1^.

**Table 1:** Reaction energies Δ*E* for various dissociation reactions in clusters including the glyoxylate anion calculated at the MP2/aug-cc-pVDZ//B3LYP/def2TZVP level of theory. Thermal energies *E*_therm_ at *T* = 298.15 K are calculated employing the vibrational frequencies calculated at the B3LYP/def2TZVP level. Values obtained at the CCSD/aug-cc-pVDZ level are given in parentheses. See Table S1 for further reaction energies

Reactants	Products	Δ*E* [kJ mol^−1^]	*E*_therm_ [kJ mol^−1^]
C_2_HO_3_·H_2_O^−^	C_2_HO_3_^−^ + H_2_O	60.9 (59.7)	20.5 (20.3)
	COOCH(OH)_2_^−^	6.8 (6.5)	
Na_4_Cl_2_(C_2_HO_3_)(H_2_O)^+^	Na_4_Cl_2_(C_2_HO_3_)^+^ + H_2_O	58.5	51.8
Na_5_Cl_3_(C_2_HO_3_)(H_2_O)^+^	Na_5_Cl_3_(C_2_HO_3_)^+^ + H_2_O	57.5	60.1
Na_3_(C_2_HO_3_)_2_^+^	Na_2_(C_2_HO_3_)^+^ + Na(C_2_HO_3_)	186.0	40.9
Na_3_(C_2_HO_3_)_2_(H_2_O)^+^	Na_3_(C_2_HO_3_)_2_^+^ + H_2_O	46.9	48.7
	Na_2_(C_2_HO_3_)^+^ + Na(C_2_HO_3_) + H_2_O	232.9	
Na_3_(C_2_HO_3_)_2_(H_2_O)_2_^+^	Na_3_(C_2_HO_3_)_2_^+^ + 2H_2_O	93.8	59.1

In the IRMPD spectra, the C–H vibration is clearly identified at around 2900 cm^−1^ for most cluster sizes. For *n* = 4, the noise level is relatively high, as a result of low internal energy together with a relatively high water binding energy and overall relatively low ion signal. A free O–H vibration centered around 3650 cm^−1^ is present in all spectra. For *n* = 4, 5, another weak transition appears at 3550 cm^−1^ which is also assigned to an O–H vibration. Assuming that intact water molecules are present, these two vibrations are assigned as asymmetric and symmetric stretching modes of the H_2_O molecule, in agreement with the calculations (Fig. 2). This implies that the water molecule binds with the oxygen atom to Na^+^, as in the low-lying isomers IVa,b, Va,b. For larger clusters, a broad band in the range of 3100–3600 cm^−1^ is observed. This feature is attributed to the interaction of an OH group with a chloride or glyoxylate anion, *e.g.* isomers VIb and VIIb (Fig. S1†). For these larger clusters, the broad redshifted bands due to donor hydrogen bonds of the water molecule to negative charge centers overlap with the symmetric stretch of free H_2_O so that only the stronger asymmetric stretch at higher frequencies can be identified in the experimental data. However, isomers featuring a water molecule bound only to Na^+^ ions are probably also present.

Overall, the calculated position of IR transitions help explain the experimental measurements. The position of the C–H vibration is captured reliably by nearly all the isomers and the position of the O–H absorptions does not change significantly with increasing cluster size. Similar features are also observed in Na_4_Cl(C_2_HO_3_)_2_(H_2_O)_*m*_^+^ and Na_5_Cl_2_(C_2_HO_3_)_2_(H_2_O)_*m*_^+^, *m* = 1, 2 (Fig. S4†). However, the calculated spectra are very similar for glyoxylate-doped clusters with a bound water molecule and the isomeric *gem*-diol, and thus do not allow for an unambiguous assignment. However, the *gem*-diol isomers are always higher in energy, and the equilibrium population is thus expected to lie largely on the side of glyoxylate and water.

### UV spectroscopy of hydrated glyoxylate

**Fig. 3:**
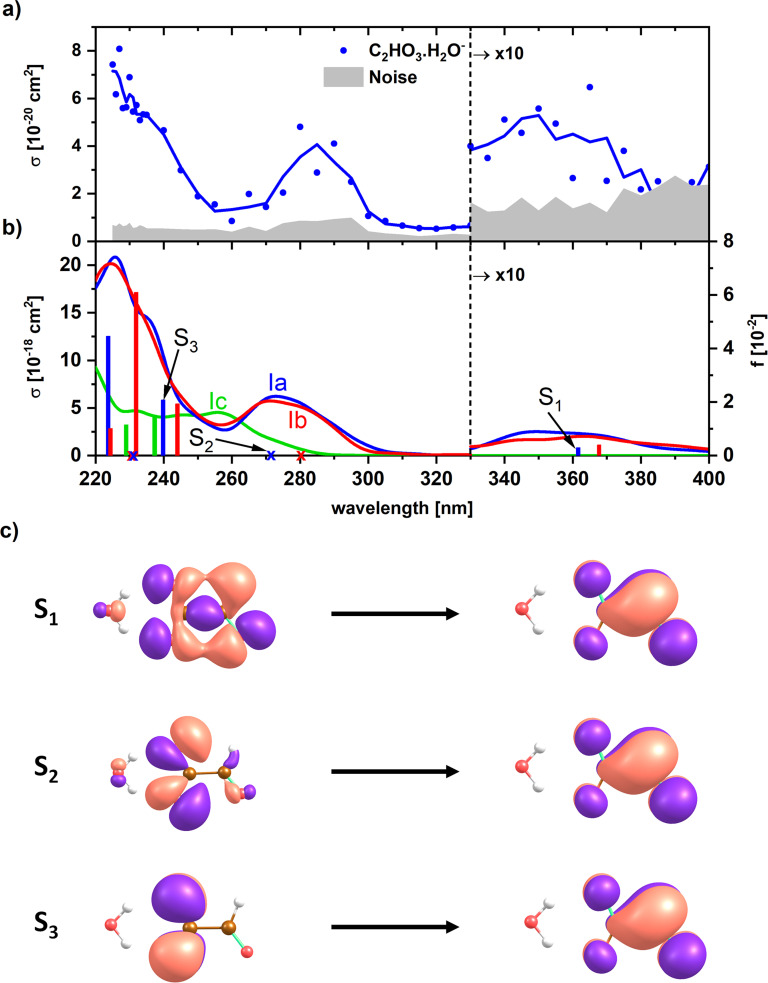
(a) Experimental photodissociation spectrum of [C_2_HO_3_·H_2_O]^−^. (b) Calculated electronic transitions in glyoxylate with water and *gem*-diol, isomers Ia, Ib and Ic (see Fig. 1). Excited states were calculated at the EOM-CCSD/aug-cc-pVDZ//CCSD/aug-cc-pVDZ level of theory. Spectra were modeled using reflection principle with the ground state potential energy surface sampled at the B3LYP/def2TZVP level. The modeled spectra were empirically shifted by −0.3 eV to match the position of the bands to the calculations in the CCSD minimum; no shift was applied for the calculated transitions in the minimum structures. (c) Natural transition orbitals corresponding to the excitation into selected states of isomer Ia as calculated at the TD-CAMB3LYP/aug-cc-pVDZ//CCSD/aug-cc-pVDZ level.

For a less ambiguous structural assignment of glyoxylate *vs. gem*-diol in the presence of water, UV photodissociation spectra were measured. Since the aldehyde functional group is responsible for the absorption of glyoxylate in the actinic region, we expect the absorption to disappear for *gem*-diol. Fig. 3 shows the experimental UV spectrum for the simplest anionic complex, [C_2_HO_3_·H_2_O]^−^, along with calculations for both the glyoxylate anion with attached water, C_2_HO_3_^−^·H_2_O, and the *gem*-diol form, COOCH(OH)_2_^−^. The only dissociation reaction observed in the experiment is evaporation of one water molecule, requiring about 60 kJ mol^−1^ for a glyoxylate anion with attached water molecule (Table 1).

Although we do not observe any evidence for bond photolysis in the glyoxylate moiety, we cannot strictly rule it out. In the case of homolytic C–C bond photolysis, one would expect formation of a (CO_2_^−^)H_2_O fragment, which due to its small electron affinity of only 0.16 eV (ref. 59) most likely undergoes electron detachment before it can be detected by FT-ICR mass spectrometry, which requires a lifetime of >0.1 s. A similar argument applies to heterolytic bond photolysis, which was recently reported in photoelectron spectroscopy of pyruvate by Verlet and co-workers.^36^ In the case of glyoxylate, the potential heterolytic photolysis product HCO^−^ has an electron affinity of 0.31 eV,^60^ and again is likely to undergo either autodetachment or photodetachment by a second laser photon. We therefore may only detect those fragments caused by absorption events that proceed *via* internal conversion to the electronic ground state followed by water evaporation, and miss C–C bond photolysis. Due to the small absorption cross sections, there is also no chance of identifying these events by signal depletion of the parent ion in our experiment. For the same reasons, we are not able to provide a photodissociation or photodetachment spectrum of bare glyoxylate.

However, Wang and co-workers recently reported that one water molecule reduces the quantum yield of pyruvate photolysis, and two water molecules completely block all photochemical reaction paths.^37^ In our previous work,^45^ we have shown computationally that photodissociation to CO_2_^−^ or HCO^−^ is expected to be efficient in the S_1_ state. To explore whether hydration quenches C–C photolysis in glyoxylate in the same way as in pyruvate, we followed the C–C bond photolysis reaction path of C_2_HO_3_^−^(H_2_O), Ia, in the S_1_ state, *i.e.* we scanned the C–C bond length and optimized the structure at each fixed value of the C–C bond length in the S_1_ state. To locate potential conical intersections, we also calculated the energy of the S_0_ state in the structures optimized along the S_1_ reaction path, see Fig. S5 in ESI.† Indeed, the C–C bond photolysis is not feasible in the S_1_ state. This scan also does not provide any evidence for a conical intersection, but given the complexity of the 3*N* – 6 dimensional hypersurfaces of all excited states, we cannot strictly rule it out. If there is a conical intersection along some other coordinate that connects S_1_ and S_0_, the complete photon energy is available for statistical dissociation in the ground state. In the potential absence of a conical intersection, fluorescence is the only remaining relaxation pathway. Fluorescence near the S_1_ minimum leads to excess vibrational energy on the order of 1 eV in the S_0_ ground state, which is available for water evaporation. In summary, the observed photodissociation product can be rationalized with or without a conical intersection.

In the experimental photodissociation spectrum, three absorption bands are observed, with the most intense one in the deeper UV below 250 nm. A weak band is observed in the actinic region, peaking at ∼350 nm. At ∼280 nm, a relatively narrow band with a significant intensity is recorded, which was not observed in sodium chloride clusters doped with glyoxylate.^45^ There, the UV absorption vanished completely at 250 nm and reappeared only at 320 nm.

Finally, electron detachment cannot be excluded in the experiment. Vertical electron detachment energy is calculated as 4.06 and 4.73 eV for the bare glyoxylate and isomer Ia, respectively (see Table S4†). The channel could be thus operative below ∼260 nm in C_2_HO_3_^−^(H_2_O). However, no indication for considerable electron detachment is observed in the measured spectra, such as reduced signal intensity of the precursor or fragment ion or both. Upon electron detachment, the glyoxylate radical dissociates into CO_2_ and CHO, the adiabatic detachment energy is therefore by about 2 eV lower than the vertical one (Table S4†).

Any signal in the experimental photodissociation spectrum is necessarily caused by the absorption of at least one photon. To understand the origin of the experimental spectrum, we modeled the absorption spectrum by combining molecular dynamics with the linear reflection principle. Fig. 3b shows the modeled spectra of the glyoxylate water complex when starting molecular dynamics sampling from Ia, Ib, and the *gem*-diol Ic. The modeled spectra of both Ia and Ib isomers for the glyoxylate–water system are almost identical as both isomers easily interconvert during the dynamics runs. The spectra reproduce all experimentally observed features, their shape matches the relative intensity of bands observed in the experiment, but the intensity is predicted to be overall two orders of magnitude higher than the recorded one, which could be due to C–C bond photolysis followed by electron auto- or photodetachment. The absorption bands in the simulated spectrum lie about 0.2–0.3 eV too high in energy due to the limits in the quantum chemical methods employed (molecular dynamics at the B3LYP/def2TZVP level) as well as zero-point effects,^51^ which justifies the empirical shift employed in Fig. 3. The presence of the band at ∼280 nm that is not observed in glyoxylate-salt clusters^45^ is connected to the flexibility of the glyoxylate anion in the glyoxylate–water complex. Namely, the transition at ∼280 nm is symmetrically forbidden for planar glyoxylate and receives a considerable intensity only if the molecule may rotate along the central C–C bond. This rotation might take place in the glyoxylate–water cluster; the flexibility is more limited in a salt cluster, and the band thus disappears. First three electronic transitions correspond to promotion of an electron into a delocalized π orbital (Fig. 3c).

Additionally, we modeled the spectrum of the *gem*-diol form Ic, COOCH(OH)_2_^−^. Here, considerable intensity is observed at ∼260 nm where an absorption minimum is observed in the experiment. The absence of this band in the experiment and the observation of the peak at ∼350 nm are strong indicators for the presence of the water-attached glyoxylate form.

**Fig. 4:**
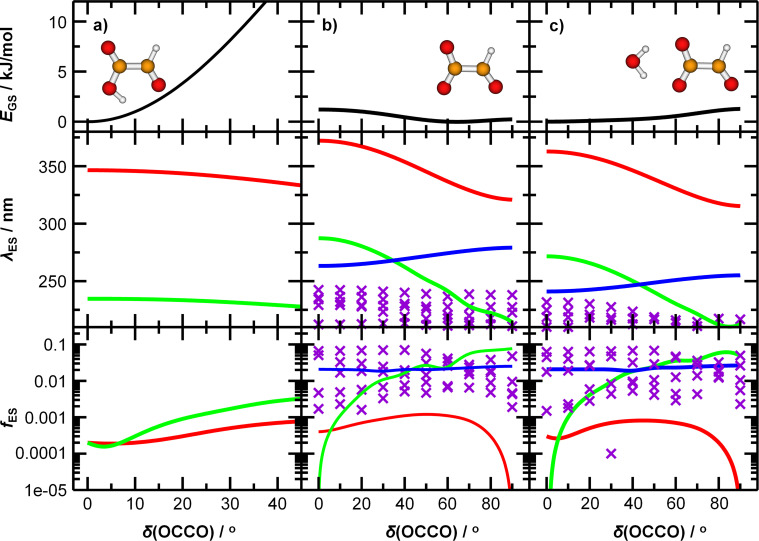
Potential energy curve of the ground state *E*_GS_, wavelength corresponding to transitions into excited states *λ*_ES_, and the respective oscillator strength *f*_ES_ for (a) glyoxylic acid, (b) glyoxylate, and (c) glyoxylate complexed with water. Calculated at the (EOM-)CCSD/aug-cc-pVDZ level of theory. Higher-lying excited states are shown as violet crosses as they are mixed and cannot be easily distinguished from each other.

To understand the origin of the peak broadening, in particular in the S_1_ ← S_0_ transition in the actinic region, we scanned the dihedral angle *δ*(OCCO) in glyoxylic acid, glyoxylate, and glyoxylate complexed with one water molecule, see Fig. 4. In the electronic ground state, the intramolecular hydrogen bond in glyoxylic acid, Fig. 4a, keeps the molecule planar while the barrier for a complete rotation in glyoxylate or the glyoxylate water complex, Fig. 4b and c, respectively, is less than 5 kJ mol^−1^. (The difference with respect to the previous calculations on glyoxylate^45^ can be traced to the diffuse basis functions used within the aug-cc-pVDZ basis set.) In our room temperature experiment, this hindered rotation will thus be possible. The position of the S_1_ ← S_0_ transition, red line in the second row, covers about 320 to 370 nm, very close to the experimentally observed range. The oscillator strength, red line in 3rd row, reaches a maximum around *δ*(OCCO) = 50°, which contributes to the observed peak maximum. The S_2_ and S_3_ state, green and blue lines, appear at significantly shorter wavelengths. Comparison of Fig. 4b and c predicts a slight blue-shift of the S_1_ absorption and a significant blue-shift of the S_2_ and S_3_ bands upon hydration.

### UV spectroscopy of sodium glyoxylate clusters doped with up to two water molecules

**Fig. 5:**
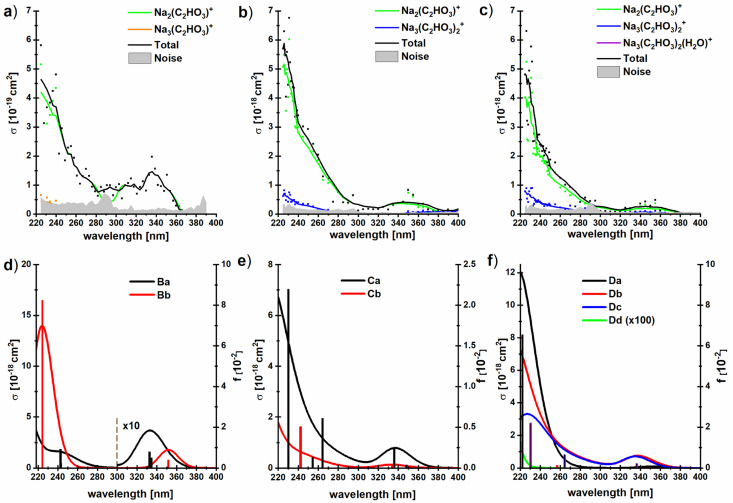
(a–c) Experimental photodissociation cross section of the clusters Na_3_(C_2_HO_3_)_2_(H_2_O)_*m*_^+^, *m* = 0–2, with partial cross sections for individual fragments; (d–f) corresponding oscillator strengths and absorption spectra modeled using linearized reflection principle at the TD-CAM-B3LYP/aug-cc-pVDZ//B3LYP/def2TZVP level of theory, see Fig. 1d–f for structures. No energy shift was applied to spectra.

To investigate if this behavior changes when glyoxylate is embedded in a salt environment, photodissociation spectra of the dry and hydrated systems Na_3_(C_2_HO_3_)_2_(H_2_O)_*m*_^+^, *m* = 0–2, were measured, Fig. 5. These ions were chosen because they are generated in high intensity in the ESI source, the fragment intensity is not distributed over many different peaks, and the presence of two chromophores is expected to double the absorption cross section. A very similar spectral shape is recorded, irrespective of the number of water molecules, with fragments coming from absorptions in the deep UV as well as in the actinic region. Interestingly, the photodissociation cross section increases by one order of magnitude upon addition of water molecules.

All hydrated sodium-glyoxylate photodissociation spectra show an absorption in the deeper UV below 290 nm, with cross sections increasing down to the 225 nm limit of the available wavelengths. A broad, intense transition in the actinic region around 350 nm was observed, which again indicates that glyoxylate does not fully convert to its *gem*-diol form. Also here, it cannot be strictly ruled out that some glyoxylate ions rearrange to the *gem*-diol form, but the data supports the results of [C_2_HO_3_·H_2_O]^−^ discussed above that the system does not necessarily rearrange to the *gem*-diol form in the presence of water.

It is remarkable that the spectrum is significantly broader in the UV region compared to the previously studied dry glyoxylate doped sodium chloride clusters where the absorption started at 250 nm, with an increase towards shorter wavelengths.^45^ One possible explanation is that the band observed at 260–280 nm for the glyoxylate–water complex in Fig. 3 now merges with the band below 250 nm due to the presence of multiple isomers. However, also the smaller cluster size and the absence of chloride ions, which are both effects increasing the conformational flexibility of the studied clusters, could be responsible for the broadening.

Our calculations within linearized reflection principle approximation reproduce the experimental spectra well, showing both the band at ∼340 nm and the rising absorption below 280 nm (Fig. 5d–f). As expected, the low-energy absorption is connected exclusively to the presence of the glyoxylate anion and disappears in the Dd isomer where two *gem*-diol units are present. Depending on the structure of the glyoxylate anion, the intensity of the low-energy band might vary considerably. In particular, we expect that its intensity rises if we account for thermally activated torsional motion along the C–C axis, as in the case of C_2_HO_3_^−^·H_2_O in Fig. 3. Due to the presence of several isomers and the usage of the simplified linearized reflection principle treatment, the energy shift between the experimental and modeled spectrum is hard to assess.

The dominant fragmentation channel observed upon excitation of Na_3_(C_2_HO_3_)_2_(H_2_O)_*m*_^+^, *m* = 1–2, is loss of a neutral sodium glyoxylate unit (Fig. 5 and Table 1), together with all water molecules. Water evaporation from the Na_3_(C_2_HO_3_)_2_(H_2_O)_1,2_^+^ clusters is observed to a lesser extent. The photon energy is fully sufficient to afford these dissociations, see Tables 1 and S1.† The potential Na^+^ product with reaction energy of 197 kJ mol^−1^ (for Na_3_(C_2_HO_3_)_2_^+^) lies outside the mass range of the instrument and cannot be detected. In our previous study with dry sea salt-glyoxylate clusters, fragments including COO˙^−^ and HCOO^−^ in the deep UV as well as in the actinic region have been observed, with small cross sections down to 10^−21^ cm^2^.^45^ The COO˙^−^ fragment does not appear in the present study, which is most likely due to the higher noise level in the present work, being in the range of 10^−19^–10^−20^ cm^2^.

It is quite intriguing that the experimental photodissociation cross section is increased by an order of magnitude upon hydration while the simulated spectra overall feature quite similar absorption cross sections with and without hydration. Moreover, the calculated cross sections agree quantitatively with experiment for the hydrated species. This implies that the quantum yield for photodissociation is relatively small without water, and the presence of water molecules significantly increases this value. Loss of Na(C_2_HO_3_) from Na_3_(C_2_HO_3_)_2_^+^ requires 186 kJ mol^−1^, and loss of one or two water molecules from the singly or doubly hydrated species needs 47 kJ mol^−1^ or 93 kJ mol^−1^, respectively, see Table 1. At the same time, Na_2_(C_2_HO_3_)^+^ is the by far dominant photodissociation product for all three species. We have previously shown that the presence of sodium ions has a significant impact on the S_0_, S_1_ potential energy surfaces, leading to a conical intersection along the C–C dissociation coordinate. We may thus assume that internal conversion *via* this or another conical intersection is the relaxation pathway of sodium glyoxylate clusters that leads to formation of the Na_2_(C_2_HO_3_)^+^, regardless of hydration, since this makes the full photon energy available for statistical dissociation in the electronic ground state.

Formation of the Na_3_(C_2_HO_3_)_2_^+^ product by loss of one or two water molecules, however, is not consistent with internal conversion, since the photon energy in the range of 310–530 kJ mol^−1^ should lead to additional fragmentation, at least at the high-energy end of the spectrum. This suggests that the Na_3_(C_2_HO_3_)_2_^+^ product arises from loss of water preceding or following fluorescence. The Na_3_(C_2_HO_3_)_2_^+^ product thus hints at a competition between internal conversion and fluorescence. The relatively small photodissociation cross section of bare Na_3_(C_2_HO_3_)_2_^+^ thus could be rationalized by a relatively high fluorescence quantum yield of this cluster ion, which would be reduced upon hydration in favor of internal conversion.

## Conclusion

Infrared multiple photon dissociation spectra of sodium chloride clusters doped with glyoxylate and water are consistent with the calculated spectra of both the hydrated glyoxylate-doped salt cluster and the *gem*-diol form, but the latter is energetically less preferred according to our DFT calculations. UV/vis spectra are consistent with the calculated energetic preference, showing the typical weak and broad absorption of glyoxylate in the actinic region for all investigated systems, which corresponds to the S_1_ ← S_0_ transition. Quantum chemical modeling reproduces the spectra very well. Hydrated glyoxylate exhibits a pronounced peak around 280 nm, assigned to the S_2_ ← S_0_ transition. Hydration changes the dihedral angle of glyoxylate from 70° to planar, but thermal energy makes all dihedral angles accessible in glyoxylate with and without attached water. In sodium glyoxylate clusters, hydration increases the photodissociation cross sections by an order of magnitude, an effect that is not mirrored in the computational modeling. A possible explanation for this behavior is that the dry clusters relax predominantly by fluorescence, while hydration shifts the quantum yield towards internal conversion. No C–C bond photolysis products are detected, which indicates that their quantum yield is well below 10%, as observed previously for glyoxylate embedded in sodium chloride clusters.

Since it proved very difficult to generate hydrated species on our instrument, the selection of studied species is rather limited. In our previous study on sodium chloride clusters doped with one glyoxylate ion, band position and intensity of the absorption in the actinic region were mostly independent of cluster size, and do not differ much from the bare hydrated ion studied here. However, some subtle influence of cluster size or charge state cannot be ruled out, and experiments on anionic or doubly charged clusters are highly desirable to get a more complete picture.

Regarding the partitioning of native glyoxylate *vs. gem*-diol in the hydrated species, we have not found any evidence for *gem*-diol form in our experiment, but we cannot rule out that a fraction of the species has been converted. With increasing number of water molecules, however, the clusters are expected to gradually reach the solution phase equilibrium of glyoxylate and its *gem*-diol form, but probing the onset of *gem*-diol formation experimentally will be extremely difficult.

## Conflicts of interest

There are no conflicts of interest to declare.

## Supplementary Material

EA-003-D3EA00039G-s001

EA-003-D3EA00039G-s002
