# Supplementary material for: Spectroscopy of cluster aerosol models: IR and UV spectra of hydrated glyoxylate with and without sea salt
Source: Environ Sci Atmos. 2023 Aug 30;3(10):1396–406. doi: 10.1039/d3ea00039g (PMC10569154; doi:10.1039/d3ea00039g)
Supplement: EA-003-D3EA00039G-s001 [file EA-003-D3EA00039G-s001.pdf]

Electronic Supplementary Material (ESI) for Environmental Science: Atmospheres.
This journal is © The Royal Society of Chemistry 2023

Supporting Information for the manuscript:

Spectroscopy of Cluster Aerosol Models: IR and UV Spectra of Hydrated Glyoxylate with and without Sea Salt

Nina K. Bersenkowitsch, Sarah Madlener, Jakob Heller, Christian van der Linde, Milan Ončák, and Martin K. Beyer**

Institut für Ionenphysik und Angewandte Physik, Universität Innsbruck, Technikerstraße 25, 6020 Innsbruck, Austria

E-mail: milan.oncak@uibk.ac.at; martin.beyer@uibk.ac.at

Table S1: Reaction energies ΔE of $Na_nCl_{n-2}(C_2HO_3)(H_2O)^+$ with n = 4-11 and $Na_n(C_2HO_3)_{n-1}(H_2O)_m^+$ for n = 3,4 and m = 1-3 (m < n) calculated at the MP2/aug-cc-pVDZ//B3LYP/def2TZVP level of theory. Thermal energy E_{therm} of the parent ion is also given.

parent ion	Products	ΔE [kJ/mol]	E_{therm} [kJ/mol]
$Na_6Cl_4(C_2HO_3)(H_2O)^+$	$Na_6Cl_4(C_2HO_3)^+ + H_2O$	53.7	72.3
$Na_7Cl_5(C_2HO_3)(H_2O)^+$	$Na_7Cl_5(C_2HO_3)^+ + H_2O$	52.8	84.1
$Na_8Cl_6(C_2HO_3)(H_2O)^+$	$Na_8Cl_6(C_2HO_3)^+ + H_2O$	55.6	93.0
$Na_9Cl_7(C_2HO_3)(H_2O)^+$	$Na_9Cl_7(C_2HO_3)^+ + H_2O$	75.0	101.8
$Na_{10}Cl_8(C_2HO_3)(H_2O)^+$	$Na_{10}Cl_8(C_2HO_3)^+ + H_2O$	51.0	114.5
$Na_{11}Cl_9(C_2HO_3)(H_2O)^+$	$Na_{11}Cl_9(C_2HO_3)^+ + H_2O$	67.0	123.1
$Na_3(C_2HO_3)_2(H_2O)_2^+$	$Na_2(C_2HO_3)^+ + NaC_2HO_3 + 2\ H_2O$	279.8	59.1

Table S2: Calculated wavenumber $\tilde{\nu}$ (in cm^{-1}) and IR intensities I (in $km.mol^{-1}$) for glyoxylate with attached water, isomer 1, and its gem-diol form, calculated at the B3LYP/def2TZVP level of theory; wavenumbers were scaled by a factor of 0.96.

$C_2HO_3^-.H_2O$		$O_2CC(OH)_2H^-$	
$\tilde{\nu}$	I	$\tilde{\nu}$	I
39	0.5	58	0.4
54	8.5	276	11.8
76	1.1	342	26.8
174	10.9	498	2.0
253	18.3	528	0.8
335	22.1	533	29.9
349	62.0	554	7.1
396	0.7	598	110.1
553	41.9	785	34.1
706	182.1	876	9.5
782	45.3	1013	35.5
904	8.6	1059	335.0
955	2.3	1183	24.0
1305	111.3	1305	142.5
1362	73.0	1318	9.9
1600	438.0	1322	218.5
1645	362.3	1432	100.4
1692	222.2	1629	423.9
2675	228.1	2789	148.8
3492	176.7	3397	14.4
3527	180.6	3406	273.2

Table S3: Lowest eight electronic excitation energies E (in eV) and the respective oscillator strength *f* in glyoxylate and five $C_2HO_3^-.H_2O$ isomers. Calculated at the EOM-CCSD/aug-cc-pVDZ//CCSD/aug-cc-pVDZ level of theory.

	E	*f*
**glyoxylate**	3.72	0.0010
	4.50	0.0221
	5.18	0.0111
	5.31	0.0464
	5.45	0.0075
	5.65	0.0114
	5.71	0.0343
	5.87	0.0303
**Ia**	3.42	0.0003
	4.56	0.0000
	5.14	0.0208
	5.35	0.0000
	5.51	0.0446
	5.67	0.0176
	5.90	0.0015
	6.12	0.0632
**Ib**	3.38	0.0004
	4.42	0.0000
	5.10	0.0193
	5.36	0.0000
	5.37	0.0609
	5.55	0.0101
	5.81	0.0026
	6.56	0.0001
**Ic**	5.22	0.0147
	5.41	0.0115
	5.74	0.0013
	5.75	0.0096
	5.81	0.0000
	6.06	0.0070
	6.13	0.0132
	6.23	0.0105
**Id**	3.41	0.0004
	4.55	0.0000
	5.06	0.0257
	5.36	0.0000
	5.49	0.0487
	5.57	0.0263
	5.81	0.0025
	5.84	0.0193
**Ie**	3.83	0.0015
	4.83	0.0156
	5.32	0.0604
	5.47	0.0351
	5.55	0.0050
	5.61	0.0197
	5.68	0.0055
	5.85	0.0089

Table S4: Vertical and adiabatic detachment energy (in eV) of the bare glyoxylate anion (**glyoxylate**) and the most stable isomer of glyoxylate complexed with water (**Ia**). Calculated at the CCSD/aug-cc-pVDZ//B3LYP/def2TZVP level of theory. The large difference between VDE and ADE is induced by dissociation of the glyoxylate anion to CO_2 + CHO upon electron detachment.

	VDE	ADE
**glyoxylate**	4.06	2.39
**Ia**	4.73	2.89

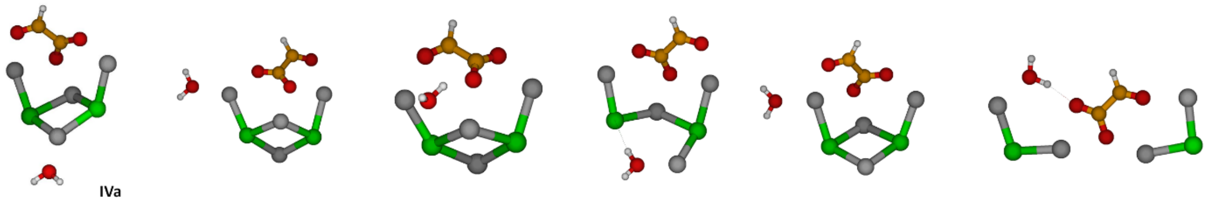

IVa

0 **2** **8** **8** **9** **101**

Figure S1: Further conformations of $Na_4Cl_2(C_2HO_3)(H_2O)^+$ in addition to the ones shown in Figure 1. Calculated at the MP2/aug-cc-pVDZ//B3LYP/def2TZVP level of theory, the relative energy is given in kJ/mol. Color code: Na grey, Cl green, C orange, O red, H white.

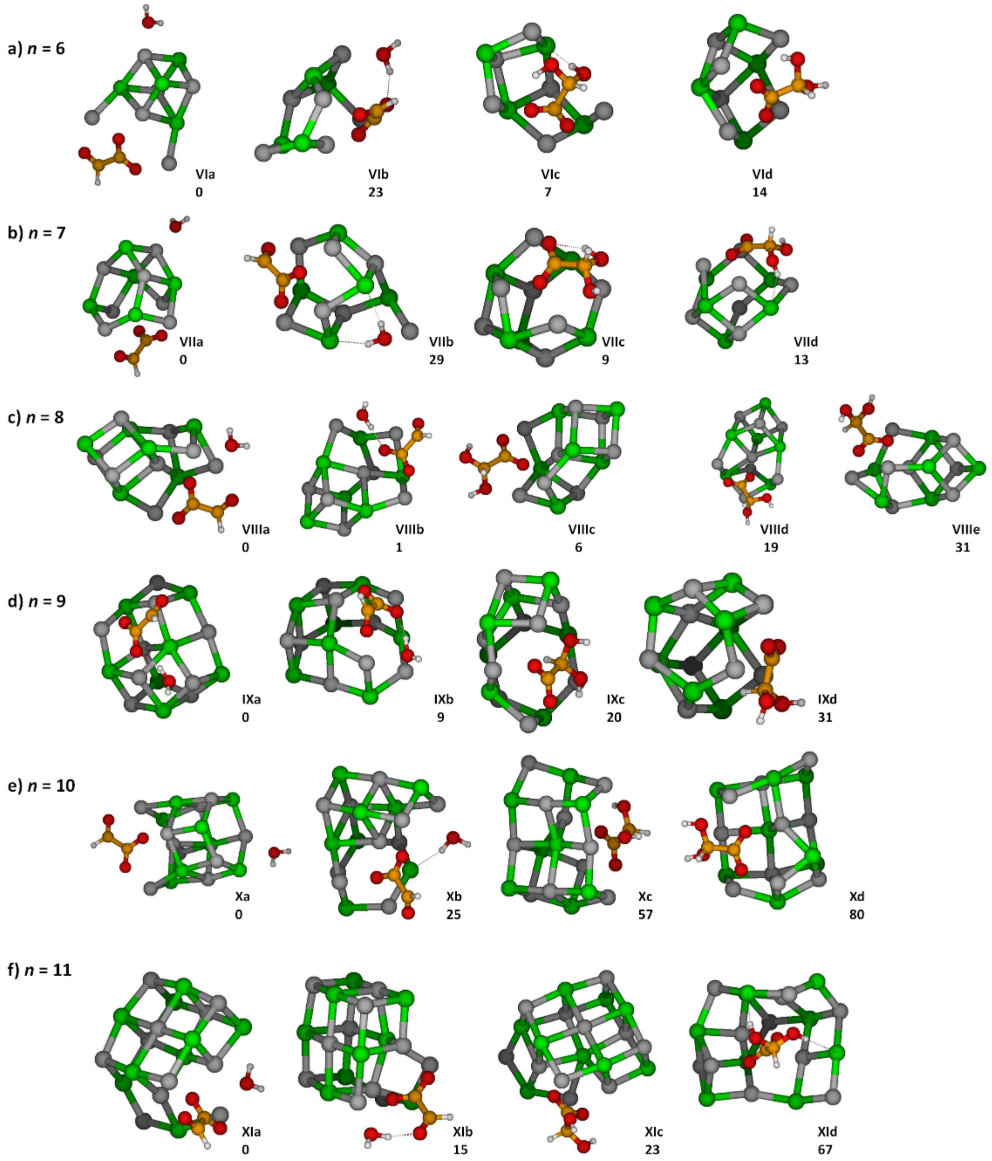

Figure S2: Calculated structures of $Na_nCl_{n-2}(C_2HO_3)(H_2O)^+$, n = 6–11. Calculated at the MP2/aug-cc-pVDZ//B3LYP/def2TZVP level of theory, the relative energy is given in kJ/mol. Color code: Na grey, Cl green, C orange, O red, H white.

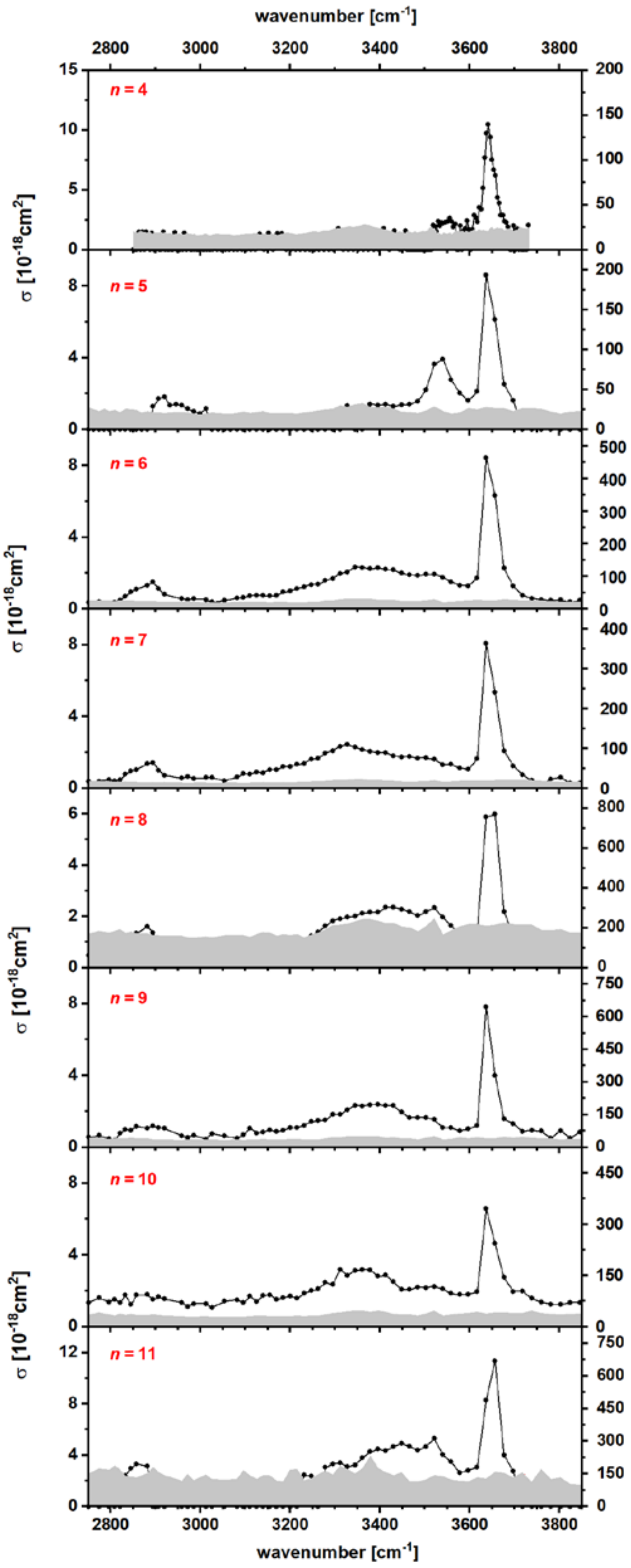

Figure S3: IR multiple photon dissociation spectra of the clusters $Na_nCl_{n-2}(C_2HO_3)(H_2O)^+$ with $n = 4$–11 with an irradiation time of 5s, analyzed assuming a 2-photon process.

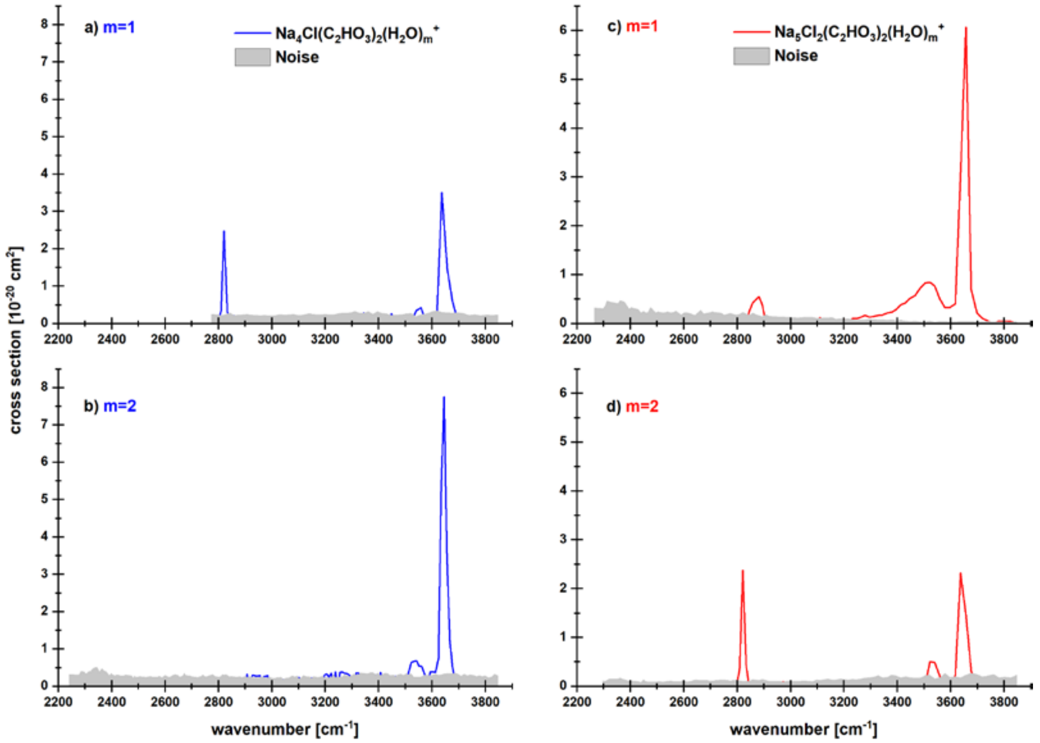

Figure S4: Experimental IRMPD cross section for the cluster $Na_4Cl(C_2HO_3)_2(H_2O)_m^+$ and $Na_5Cl_2(C_2HO_3)_2(H_2O)_m^+$ for **a,c)** $m = 1$ and **b,d)** $m = 2$ with an irradiation time of 5 s, assuming 1-photon dissociation.

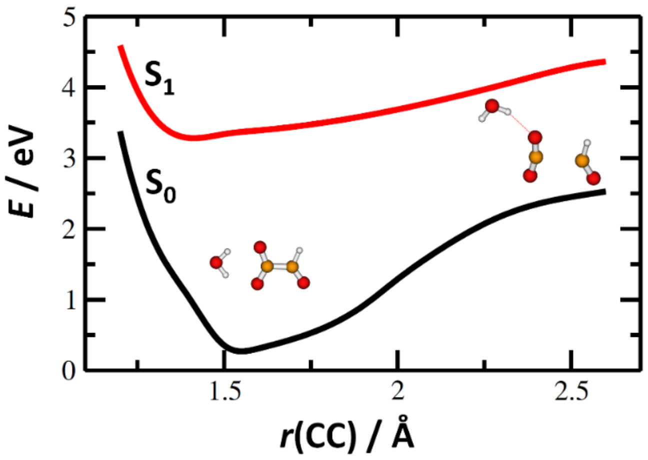

Figure S5: Dissociation curve of a glyoxylate anion with an attached water molecule as optimized in the S_1 state at the CASSCF(6,6)/def2TZVP and single-point recalculated at the MRCI(6,6)/def2TZVP level. The zero in energy is set to the MRCI energy calculated in the structure optimized at the CCSD/aug-cc-pVDZ level. The EOM-CCSD/aug-cc-pVDZ excitation energy in the minimum is calculated as 3.42 eV.

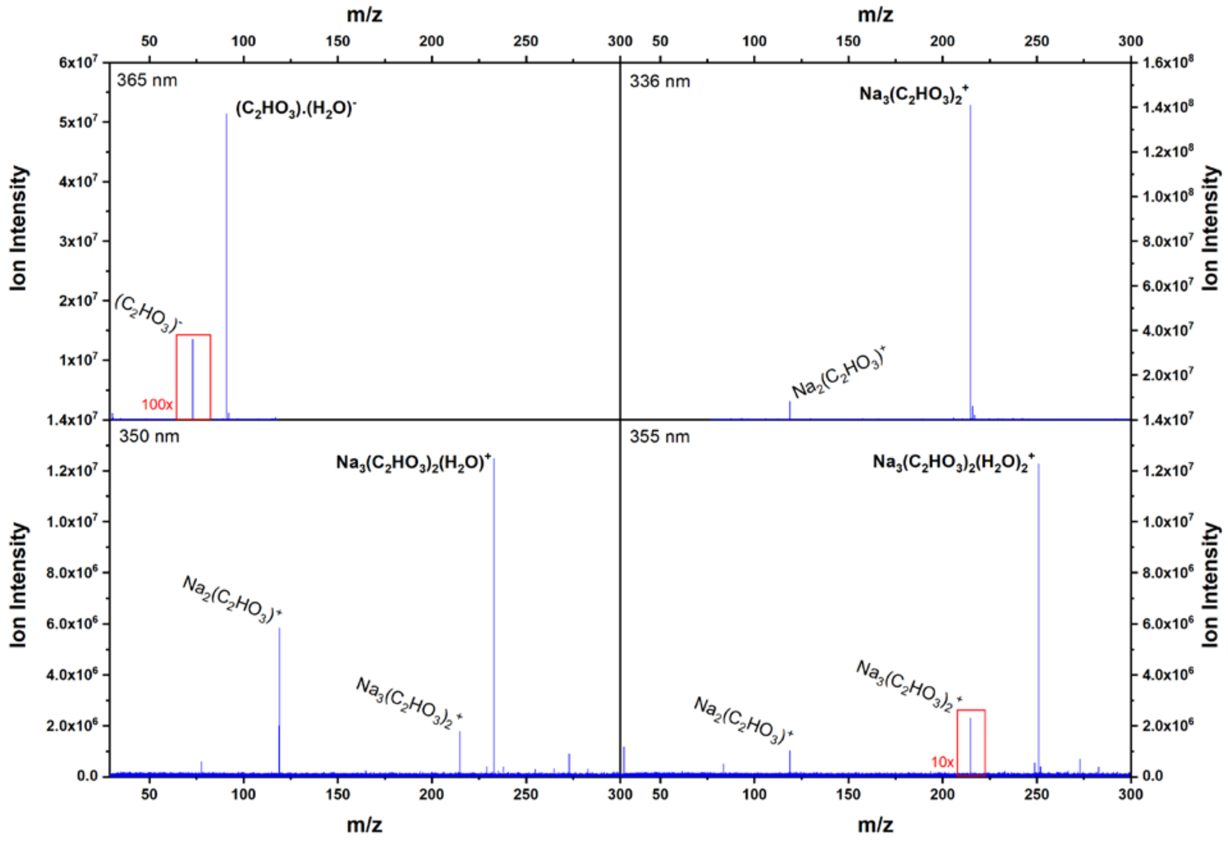

Figure S6: Selected mass spectra taken for the UV-spectra shown in Figure 3 and Figure 5 at the absorption maximum in the actinic region. Peaks not labeled are either electronic noise, 3rd harmonics of intense peaks, or isotopologues of the main peak.

Cartesian coordinates (in Å) of structures along with zero-point corrected energy (in Hartree)

CCSD/aug-cc-pVDZ

H2O
E = -76.441822
O 0.000000 -0.000000 0.116885
H -0.000000 0.765005 -0.467539
H -0.000000 -0.765005 -0.467539

C2H2O3
E = -302.345327
C 0.754147 0.758323 0.000000
O 0.149741 1.814102 0.000000
C -0.000000 -0.580921 -0.000000
O 0.584247 -1.638090 -0.000000
O -1.339303 -0.456153 -0.000000
H 1.860064 0.680569 0.000000
H -1.542424 0.496147 0.000000

C2HO3-
E = -301.816483
C 0.885009 -0.249507 0.351215
C -0.596540 0.026510 0.027668
O -0.980191 1.220788 0.041691
O 1.857124 0.110423 -0.302916
O -1.223167 -1.051370 -0.179694
H 1.039057 -0.900747 1.254057

C2HO3-.H2O, Ia
E = -378.086414
C 0.692171 1.618217 0.000000
O 1.892748 1.832628 0.000000
C -0.000000 0.223004 0.000000
O -1.263530 0.323540 0.000000
O 0.734026 -0.794060 0.000000
H -0.039879 2.465665 0.000000
H -0.612973 -2.349623 0.000000
O -1.565375 -2.553416 0.000000
H -1.883124 -1.632896 0.000000

C2HO3-.H2O, Ib
E = -378.085435
C 1.280238 0.173644 0.000000
O 1.322613 -1.047565 0.000000
C 0.000000 1.066547 0.000000
O -1.117013 0.486449 -0.000000
O 0.286360 2.291399 0.000000
H 2.225399 0.772771 0.000000
H -1.460631 -1.303996 -0.000000
O -1.481252 -2.286367 -0.000000
H -0.531859 -2.461249 -0.000000

C2HO3-.H2O, Ic
E = -378.083941
C 0.029477 0.837987 0.000000
O -0.108600 1.352536 1.145168
O -0.108600 1.352536 -1.145168
C 0.406517 -0.677738 -0.000000
O -0.108600 -1.298075 -1.166041
H 1.511579 -0.791210 -0.000000
O -0.108600 -1.298075 1.166041
H -0.326171 -0.520831 -1.715216
H -0.326171 -0.520831 1.715216

C2HO3-.H2O, Id
E = -378.082390
C 1.043605 1.028323 0.000136
O 0.136045 1.843386 -0.000179
C 0.885650 -0.521321 0.000014
O 1.992846 -1.117891 -0.000102
O -0.288330 -0.977741 0.000095
H 2.113262 1.360362 0.000526
H -1.933178 -0.193631 -0.000032
O -2.915848 -0.139678 0.000142
H -3.153312 -1.073352 -0.001034

C2HO3-.H2O, Ie
E = -378.077093
C -0.021761 -0.044545 0.258854
C 1.495748 0.068765 0.055059
O 2.169888 -0.981904 0.160049
O -0.823785 -0.554796 -0.524290
O 1.825862 1.268281 -0.157552
O -3.472845 0.094151 0.160006
H -0.408908 0.446275 1.188628
H -3.450043 1.023630 -0.092406
H -2.577938 -0.221080 -0.085399

B3LYP/def2TZVP

Na4Cl2(C2HO3)(H2O)+, IVa
E = -1948.935690
C 3.400588 -0.443275 -1.015303
C 2.475814 0.409589 -0.122849
O 2.432600 1.620840 -0.403254
O 3.191970 -1.622234 -1.187734
O 1.818672 -0.219244 0.744463
Na 0.812213 3.087863 -0.382979
Na -0.118698 0.166687 1.806201
Na 1.365936 -2.410077 0.071083
Cl -1.153552 -1.982980 0.657222
Cl -1.398551 1.998389 0.393041
Na -2.731456 -0.163475 -0.406640
H 4.217746 0.084801 -1.530423
O -4.719356 -0.562650 -1.425121
H -5.115047 -1.435275 -1.536331
H -5.374394 0.065974 -1.749955

Na4Cl2(C2HO3)(H2O)+, IVb
E = -1948.931548
C -2.940650 1.261040 -0.726431
C -1.617894 1.487798 0.037675
O -1.135665 2.631305 -0.044207
O -3.321428 0.149168 -1.008998
O -1.145719 0.480297 0.621514
Na 0.982649 3.105002 0.265267
Na 0.687871 -0.557925 1.334514
Na -1.869025 -1.550818 -0.229255
O -0.795854 -2.505117 1.824132
Cl 2.581009 1.059629 0.315767
H -3.491039 2.162961 -1.035732
H -1.140940 -2.920620 2.624367
H -0.257806 -3.168593 1.363009
Cl 0.702129 -2.274725 -0.826755
Na 2.709144 -0.810383 -1.486147

Na4Cl2(C2HO3)(H2O)+, IVc
E = -1948.922760
C -3.122013 -0.153613 0.104544
C -1.755214 0.495642 0.417515
O -1.585064 1.668935 0.027139
O -2.798590 -1.341594 -0.668755
O -0.916636 -0.236866 1.006772
Na 0.157482 2.950764 -0.279132
Na 1.245092 -0.113407 1.548661
Na -0.820582 -2.307705 -0.050326
Cl 2.410679 1.705522 -0.011515
Na 3.270801 -0.446924 -1.208332
Cl 1.805344 -2.202448 -0.013230
O -3.965971 0.675506 -0.590924
H -3.603971 -1.592972 -1.139448
H -3.439471 1.425665 -0.911331
H -3.616226 -0.474986 1.025659

Na4Cl2(C2HO3)(H2O)+, IVd
E = -1948.919505
C 2.539033 -0.890762 -0.053425
C 1.141644 -1.540562 0.037676
O 0.766140 -2.167558 -0.969653
O 2.742024 0.041939 0.979009
O 0.482297 -1.355896 1.101002
Na -1.343455 -2.595536 -0.329017
Na -0.989165 0.249397 1.624151
Na 2.256664 1.951775 -0.314188
Cl -0.284256 2.378950 0.161939
Cl -2.933487 -0.540472 0.008578
Na -2.473584 1.743441 -1.101219
O 2.627147 -0.136448 -1.256404
H 2.263007 -0.685892 -1.966377
H 2.135698 -0.221478 1.690786
H 3.322949 -1.654959 -0.037336

Na4Cl2(C2HO3)(H2O)+, further isomer
E = -1948.935244
C 0.650440 3.097140 -0.535529
O 1.810885 3.026190 -0.870432
C -0.021555 1.965577 0.268665
O 0.756349 1.232075 0.932649
O -1.250041 1.860948 0.120828
Na -2.735072 0.236106 -0.107225
Na 0.508205 -0.899346 1.552669
Na 2.863360 1.093452 -0.034264
Cl -1.345883 -2.014275 0.027518
Cl 2.604203 -1.529685 0.005821
Na 0.769359 -2.892260 -1.196779
H 0.008231 3.938086 -0.840018
H -5.536689 1.201214 -0.179837
O -5.001363 0.399284 -0.193147
H -5.627291 -0.333727 -0.223316

Na4Cl2(C2HO3)(H2O)+, further isomer
E = -1948.928358
O -0.165104 2.727484 -0.745377
C -0.949669 1.919826 -0.221396
O -0.773923 1.247974 0.829642
H -2.603826 2.541874 -1.613196
C -2.244449 1.687630 -1.018873
O -2.796805 0.611555 -1.058035
Na 1.904114 2.560513 0.028476
Cl -0.003127 -2.415409 -0.547057
O -2.091140 -0.761062 1.896585
H -2.604706 -0.780902 2.711527
H -1.914826 0.179631 1.659270
Na 0.425796 -0.638927 1.408269
Na 2.416428 -1.833150 -1.193870
Na -2.442266 -1.531671 -0.290788
Cl 2.800718 0.161447 0.419323

Na4Cl2(C2HO3)(H2O)+, further isomer
E = -1948.931912
Cl 1.086721 -2.303622 0.458624
O 3.784038 0.471328 -1.012726
H 4.562427 0.969989 -1.281692
H 3.149035 1.127798 -0.648192
Cl 1.560263 2.204955 0.357608
Na 0.491717 0.110097 1.438441
Na -0.713033 3.222686 -0.254469
O -2.350031 1.757899 -0.265686
C -2.390780 0.540412 -0.022544
O -1.607476 -0.147028 0.682760
Na -1.537016 -2.380984 0.118638
O -3.450991 -1.443508 -0.878805
C -3.515342 -0.244789 -0.732216
H -4.349964 0.347676 -1.137338
Na 3.219441 -1.647378 -0.801202

Na4Cl2(C2HO3)(H2O)+, further isomer
E = -1948.933450
C 1.394502 2.713733 -0.586528
C 0.228088 2.069545 0.189296
O -0.882740 2.609928 0.028011
O 2.366192 2.070871 -0.906345
O 0.507615 1.038018 0.847959
Na -2.932231 1.903642 -0.142755
Na -0.735021 -0.689398 1.510292
Na 2.329544 -0.171316 -0.077534
O 4.404753 -1.044049 0.242407
Cl -2.974790 -0.682471 0.057759
Cl 0.659565 -2.307275 -0.056298
Na -1.630183 -2.535538 -1.201040
H 1.267956 3.767485 -0.881197
H 5.274779 -0.629447 0.233621
H 4.560805 -1.991462 0.331297

Na4Cl2(C2HO3)(H2O)+, further isomer
E = -1948.905421
C 1.135454 1.618067 0.000277
O 2.323233 1.395527 0.000236
C 0.073463 0.497522 0.000377
O 0.471448 -0.696229 0.000476
O -1.109280 0.883872 0.000379
Na -1.849553 -1.398923 0.000525
Na 2.564994 -1.511172 -0.000194
Na 4.670141 1.470441 0.000001
Cl 5.009125 -0.979033 -0.000423
O -3.578999 2.283223 -0.000286
Na -5.204605 0.741205 -0.000775
Cl -4.341000 -1.595912 0.000030
H 0.733677 2.643757 0.000242
H -2.664719 1.945052 -0.000005
H -3.522554 3.243510 0.000943

Na5Cl3(C2HO3)(H2O)+, Va
E = -2571.601077
C -0.672291 1.876169 -1.140920
O -1.703818 1.951150 -0.509707
C 0.702295 1.818123 -0.442505
O 1.689850 1.593432 -1.174205
O 0.717732 1.973591 0.804808
Na -1.262107 1.546756 1.851808
Na 2.080515 -0.581547 -1.929785
Na 2.991183 1.369304 0.859680
Na -2.727040 -1.331858 -0.392956
Cl 3.032034 -1.178482 0.476755

Cl -1.638636 -1.005014 2.005481
Cl -0.364752 -1.458796 -1.568433
Na 0.567300 -2.003617 0.918757
H -0.659563 1.843631 -2.239116
O -4.039149 0.377287 -1.076082
H -3.550071 1.212573 -1.116067
H -4.922625 0.543906 -1.419969

Na5Cl3(C2HO3)(H2O)+, Vb
E = -2571.606638
C -3.883917 -2.043560 -0.422027
O -3.340188 -3.117560 -0.317303
C -3.112357 -0.711374 -0.264443
O -1.867714 -0.805369 -0.123729
O -3.820243 0.311349 -0.281906
Na -3.265348 2.380372 0.106377
Na 0.120913 0.354458 0.170627
Na -1.051037 -2.957743 0.121007
Cl -0.817216 2.949168 0.540123
Cl 1.296160 -2.077805 0.656537
Na 3.524263 -0.970332 -0.067392
O 5.660764 -1.763038 -0.094602
H -4.963118 -1.954533 -0.621771
H 6.444856 -1.326946 -0.447285
H 5.963181 -2.610925 0.250690
Na 1.549955 3.655482 -0.145014
Cl 2.568443 1.383599 -0.641610

Na5Cl3(C2HO3)(H2O)+, Vc
E = -2571.594405
C -2.145450 0.698216 -1.395186
O -1.756424 -0.588816 -1.859064
O -3.118679 0.510310 -0.405503
C -0.927769 1.459077 -0.812265
O -1.056969 2.050521 0.289445
O 0.112004 1.435399 -1.510655
Na -2.287971 0.842037 1.775960
Na 2.085044 0.193023 -1.645116
Na 1.111535 2.795989 0.377072
Na -1.155528 -2.343370 -0.356652
Cl 1.333247 -2.226917 -1.077181
Cl 2.885878 0.993220 0.737090
Na 1.709065 -1.288089 1.428424
Cl -0.901011 -1.324004 2.059506
H -0.980268 -0.477772 -2.428451
H -3.959279 0.278079 -0.818737
H -2.552132 1.282026 -2.228508

Na5Cl3(C2HO3)(H2O)+, Vd
E = -2571.592940
C 3.734889 -0.326065 0.747806
O 4.899562 0.381044 0.562008
O 3.748157 -1.554555 -0.020602
C 2.478509 0.452583 0.292020
O 1.395422 -0.187970 0.272211
O 2.654667 1.646857 -0.026113
Na 1.206609 3.192435 -0.530448
Na 1.650086 -2.389440 -0.333485
Na -0.876427 -0.026928 -0.007887
Cl -1.286028 2.638701 -0.554502
Na -3.684619 2.222778 0.250829
Cl -3.531756 -0.258768 0.828729
Na -3.297927 -2.693446 0.056242
Cl -0.877858 -2.681037 -0.753120
H 4.678454 1.171329 0.042845
H 4.664329 -1.859979 -0.034551
H 3.645329 -0.594109 1.806123

Na6Cl4(C2HO3)(H2O)+, VIa
E = -3194.274531
C 4.773248 -0.973142 -0.168594
C 3.689242 0.014466 0.311579
O 3.941735 1.218040 0.120575
O 4.500095 -2.117830 -0.447650
O 2.648020 -0.502338 0.786892
Na 2.673784 2.935153 -0.310386
Na 2.238568 -2.626064 -0.048226
Na 0.520682 0.146867 1.219745
Cl 0.189218 2.235790 -0.610501
Cl -0.230850 -1.875594 -0.567546
Na -2.394115 2.177577 0.528640
Cl -1.798157 0.410259 2.354510
Na -2.778486 -1.366642 0.614282
H 5.789687 -0.569617 -0.294667
Na -0.958651 0.217591 -2.090314
Cl -3.384093 0.566382 -1.287104
O -4.778486 -2.155908 -0.088176
H -5.509858 -2.768852 0.035688
H -5.059308 -1.500715 -0.742305

Na6Cl4(C2HO3)(H2O)+, VIb
E = -3194.267352
C 0.967680 -2.881201 -0.755258
O 0.117807 -2.927147 0.102337
C 1.380526 -1.571444 -1.432553
O 0.500190 -0.813443 -1.904764
O 2.612573 -1.349420 -1.416358
Na -1.690758 -0.390859 -1.655558
Na 2.673170 0.576084 1.789106
Na -1.241854 -1.888275 1.641083
Na 2.309247 0.840291 -2.160039
Cl 2.304613 2.393439 -0.110461
Cl -3.343640 -1.462990 0.133123
Na -4.233694 0.942959 0.311953
Cl -2.277278 2.099314 -0.912176
Na -0.201441 2.385632 0.671521
Cl 0.081813 0.275959 2.290735
H 1.543356 -3.776385 -1.043218
O 3.918793 -1.139383 1.022068
H 3.657816 -1.397130 0.116801
H 4.739693 -1.596867 1.227542

Na6Cl4(C2HO3)(H2O)+, VIc
E = -3194.262924
C 0.147089 2.593071 0.009717
O -1.256280 2.598113 -0.127920
O 0.762443 2.147729 -1.179541
C 0.537971 1.699761 1.224176
O 1.751072 1.388289 1.316747
O -0.392028 1.348256 1.978915
Na -1.698126 -0.392256 2.298826
Na 2.226263 -0.788253 1.795921
Na 3.017617 1.612081 -0.591662
Na -2.567002 1.269269 -1.515838
Cl 3.537086 -0.932376 -0.505538
Na 1.333168 -1.950073 -1.359034
Cl -3.545896 -0.237178 0.425848
Na -2.111138 -2.111081 -0.634526
Cl -0.007851 -2.219242 1.075801
Cl -0.719564 -0.543919 -2.329298
H 0.514423 3.611314 0.166766
H 0.338540 1.316495 -1.480098
H -1.577774 2.275688 0.737983

Na6Cl4(C2HO3)(H2O)+, VId
E = -3194.264067
C -1.403840 2.658655 0.292147

O -0.700203 2.808708 -0.927486
C -0.618548 1.709925 1.239850
O -1.329274 1.011297 1.990347
O -2.663649 2.094211 -0.013188
O 0.630401 1.721179 1.159364
Na 1.475194 1.780589 -0.994046
Na -1.548456 -1.195718 2.082107
Na 2.123936 0.232782 1.959828
Na -2.222675 0.419412 -1.634411
Cl -2.948703 -1.617068 -0.119685
Na -0.746120 -2.670256 -0.904829
Cl 0.902531 -2.161382 1.182254
Cl 3.660110 0.581717 -0.135160
Na 2.525947 -1.536528 -0.981571
Cl 0.293387 -0.537070 -2.212413
H -1.008811 3.608104 -1.372180
H -2.936145 1.603904 0.783264
H -1.529524 3.634931 0.771864

Na7Cl5(C2HO3)(H2O)+, VIIa
E = -3816.952883
C 3.898053 -0.119107 -1.240920
O 4.084548 -0.061036 -0.045853
C 2.488789 -0.124464 -1.820993
O 1.937157 -1.239296 -1.963709
O 1.974627 0.988350 -2.075114
Na 1.115003 -2.887971 -0.686779
Na 1.201202 2.780684 -0.974771
Na 2.769920 0.061486 1.871770
Na -0.189277 -0.108240 -2.332398
Cl 1.398783 -2.189610 1.818531
Cl 1.471093 2.337633 1.590051
Na -1.139375 2.114297 1.626200
Cl -2.758269 0.165471 2.163465
Na -1.201591 -1.876376 1.828748
Na -3.240900 0.033045 -0.434020
O -5.205504 -0.147677 -1.591569
Cl -1.387871 1.988035 -1.136514
Cl -1.452297 -2.039994 -0.936067
H 4.742828 -0.170197 -1.949046
H -5.617631 -0.969462 -1.881846
H -5.852124 0.546065 -1.764829

Na7Cl5(C2HO3)(H2O)+, VIIb
E = -3816.940007
C -4.093411 -0.013070 -0.647827
C -2.791055 -0.027022 -1.439011
O -2.298390 -1.147703 -1.698974
O -4.093633 0.016667 0.563231
O -2.293315 1.082686 -1.733974
Na -1.339423 2.862277 -0.766592
Na -1.344379 -2.891305 -0.668938
Na -2.455026 0.047667 2.217000
Na -0.196159 -0.043427 -2.331445
Cl -1.120795 -2.188334 1.854239
Cl -1.108769 2.262638 1.782091
Na 1.424969 -1.886496 1.322461
Cl 3.162691 0.031911 1.843502
Na 1.436637 1.943134 1.259403
O 3.585378 -0.040722 -1.947479
Cl 1.159270 2.101551 -1.420084
Cl 1.154476 -2.158764 -1.348895
H -5.038178 -0.032559 -1.217680
H 2.987216 -0.809300 -1.973697
H 2.986807 0.726428 -1.996817
Na 4.834636 -0.010439 -0.108047

Na7Cl5(C2HO3)(H2O)+, VIIc
E = -3816.942604
C -3.622862 -0.218626 -0.427189
C -2.464829 0.327828 -1.296373
O -2.355105 1.569905 -1.308423
O -3.443655 -1.582977 -0.200338
O -3.605944 0.444258 0.841372
O -1.724711 -0.501983 -1.880548
Na -0.779504 2.959493 -0.619044
Na 0.239529 0.091334 -2.714203
Na -1.214076 -2.414205 -0.719143
Na -1.781717 0.034803 2.328744
Cl -0.554315 -2.216860 1.841593
Na 1.949049 -2.220104 1.160388
Cl 3.598819 -0.200265 1.342796
Na 3.391021 -0.094809 -1.233164
Na 1.992677 1.845286 1.377295
Cl -0.516566 2.319806 1.921005
Cl 1.636604 1.987975 -1.439249
Cl 1.495365 -1.992634 -1.584985
H -4.579277 -0.021548 -0.923788
H -4.229184 -1.949014 0.224935
H -3.615268 1.395577 0.654383

Na7Cl5(C2HO3)(H2O)+, VIId
E = -3816.936378
C 3.534513 -0.224629 0.380030
O 3.548403 0.854156 -0.555608
C 2.346992 -0.013358 1.360798
O 1.744169 -1.034112 1.778864
O 3.485105 -1.430723 -0.319002
O 2.082622 1.172473 1.659136
Na 0.506972 2.693610 1.445665
Na -0.280861 -0.687890 2.611329
Na 1.402458 -2.681049 0.273415
Na 1.806387 1.109689 -2.202322
Cl 0.259389 2.950013 -1.163638
Na -2.185540 2.092128 -0.842289
Cl -3.463840 -0.069048 -1.523422
Na -3.388433 -0.722321 0.985725
Na -1.578992 -1.849475 -1.784025
Cl 0.975777 -1.419416 -2.197082
Cl -1.845824 1.351637 1.868037
Cl -1.342925 -2.500070 0.891274
H 4.463602 -0.231928 0.959055
H 2.955684 -1.311083 -1.129664
H 3.403575 1.652002 -0.021817

Na8Cl6(C2HO3)(H2O)+, VIIIa
E = -4439.615341
C 4.034948 1.405133 -1.583368
C 2.509131 1.357190 -1.456620
O 1.959466 2.465389 -1.533257
O 4.731342 0.891470 -0.733842
O 1.995131 0.242666 -1.158703
Na 0.631777 3.366195 0.025743
Na 1.544802 -2.011607 -1.474086
Na 3.364866 -0.027325 0.959084
Na -0.086945 0.191739 0.093131
Cl -1.087857 -1.555507 -1.775106
Cl -1.751350 2.325535 -0.541307
Cl 1.804515 -2.108955 1.341165
Na -3.554720 1.201997 1.111325
Cl -4.675061 -0.328352 -0.691305
Na -2.774177 0.504183 -2.285108
Na -2.950702 -2.146582 0.080372
Cl -1.932015 -0.690250 2.108716
Na 0.482657 -0.661171 3.156277
Cl 1.435550 1.609933 1.991832

H 4.465637 1.999876 -2.404152
O 2.861109 -3.834785 -1.220125
H 2.897398 -3.973848 -0.263231
H 3.268699 -4.600497 -1.637305

Na8Cl6(C2HO3)(H2O)+, VIIIb
E = -4439.613109
C -4.506946 -0.972054 -1.256256
O -4.996818 -0.248787 -0.416589
C -2.987180 -1.178087 -1.310555
O -2.266445 -0.155906 -1.108339
O -2.636172 -2.358826 -1.424824
Na -1.105473 -3.178117 -0.022260
Na -0.736968 3.286223 -0.961166
Na -3.280054 0.590334 0.993458
Na -0.102694 0.000112 -0.014714
Cl -1.283488 2.291003 1.440872
Cl 1.270861 -2.285421 -0.827927
Na -0.227871 0.541693 3.213280
Cl 2.148616 0.262651 2.164259
Na 3.293061 -1.761546 0.859607
Cl 4.682955 -0.301511 -0.818785
Na 2.607346 -0.510885 -2.385292
Na 3.387172 1.683871 0.286402
Cl 1.393358 1.786069 -1.536950
Cl -1.614873 -1.391603 1.988283
H -5.126764 -1.578268 -1.935370
O -2.451667 2.408617 -2.107624
H -2.506523 1.449772 -1.904440
H -2.929150 2.559805 -2.928561

Na8Cl6(C2HO3)(H2O)+, VIIIc
E = -4439.608383
C 4.192073 -1.249249 0.988088
C 2.746189 -1.294996 0.446200
O 2.525527 -2.090543 -0.481678
O 4.687173 0.024457 0.489054
O 1.942069 -0.440413 0.922682
Na 1.172322 -1.856109 -2.245668
Na 1.100860 0.833196 2.623837
Na 3.052321 1.446048 -0.335639
Na -0.182719 0.138022 -0.098594
Cl -1.326523 -0.217517 2.411315
Cl -1.302946 -2.091854 -1.274317
Cl 1.149401 2.841716 0.871935
Na -3.437694 -0.521162 -1.773343
Cl -4.673001 -1.013251 0.481595
Na -2.512603 -2.321232 1.154679
Na -3.441502 1.115457 1.391727
Cl -2.341358 1.798031 -0.967356
Na -0.072582 3.025661 -1.507722
Cl 1.353741 0.867123 -2.353087
O 4.958960 -2.296484 0.547546
H 4.514725 -2.666336 -0.234599
H 5.651549 -0.010047 0.534806
H 4.223566 -1.225200 2.079735

Na8Cl6(C2HO3)(H2O)+, VIIId
E = -4439.609201
C -3.994296 -1.645611 -0.081210
O -3.601006 -2.083343 -1.366338
C -2.730704 -1.359446 0.754392
O -1.812087 -0.763183 0.113538
O -4.647350 -0.374916 -0.159576
O -2.751181 -1.612695 1.962495
Na -1.077992 -0.362920 2.903719
Na -1.559562 -0.968120 -2.186093
Na -3.034773 1.409140 -0.037623
Na 0.236930 0.299231 0.051894
Cl -1.359696 1.787264 -2.027423
Cl 1.058027 -1.533579 -1.874105
Cl 1.432168 -0.985899 2.188892
Na 0.013141 3.395247 -0.349772
Cl 2.326231 2.092957 -0.348436
Na 3.534801 0.578472 1.531451
Cl 4.590640 -1.109805 -0.171840
Na 2.439927 -2.546753 0.218421
Na 3.183170 0.129318 -2.006581
Cl -1.205401 2.152646 1.748426
H -4.319022 -2.571037 -1.787599
H -5.604705 -0.480276 -0.202276
H -4.638824 -2.375938 0.406490

Na9Cl7(C2HO3)(H2O)+, IXa
E = -5062.282467
C 2.681677 -2.512366 -2.224958
C 1.891916 -2.739813 -0.927719
O 0.787568 -3.311274 -1.067137
O 3.243362 -1.462845 -2.435112
O 2.392075 -2.262341 0.114474
Na 2.074698 -1.924980 2.281290
Na 2.828427 0.106670 -0.673863
Na -2.168299 -0.438229 -2.370072
Na -1.059492 -2.360508 0.054415
Cl -3.504036 -0.987334 -0.026820
Cl 0.082533 0.097739 -0.833654
Cl -0.559628 -1.742930 2.649097
Cl -2.403386 2.210725 -2.510330
Na 0.165137 2.538130 -2.024398
Cl 2.662964 2.832733 -1.144416
Na 3.478912 3.063542 1.259425
Cl 2.732329 0.715644 2.124493
Na -3.382541 1.771380 -0.111538
Cl -2.309033 1.938027 2.275934
Na -2.995950 -0.584051 2.574244
Na 0.020550 0.820259 1.825811
H 2.656378 -3.317391 -2.978328
O -1.440442 -2.683065 -2.482443
H -0.530619 -3.012531 -2.321843
H -1.933300 -3.413387 -2.873657

Na9Cl7(C2HO3)(H2O)+, IXb
E = -5062.287146
H 3.629569 -0.819381 -3.757022
C 3.489637 -0.522027 -2.704375
O 3.870550 0.552648 -2.297638
C 2.726314 -1.496417 -1.809175
O 3.283050 -1.904673 -0.759452
O 1.570100 -1.780106 -2.184410
Cl -1.264018 -3.017089 -0.443807
Na 1.397115 -3.061393 0.029018
Cl 1.081539 -1.871265 2.415650
Na 3.217080 1.692047 -0.364058
Na 2.804488 0.067846 2.692753
Cl 2.385048 2.555793 2.006011
Cl 0.593294 1.688475 -1.344931
Na -1.510475 -2.137939 2.127245
Na 0.060073 3.078343 0.997307
Cl -2.455835 3.457784 0.395621
Na -3.831101 1.258708 0.584726
Na -1.826858 2.596520 -1.994215
Cl -3.880868 -1.074402 1.737942
Na -0.479758 -0.823391 -1.925149
Cl -3.055901 0.224247 -1.929026
Na -3.714493 -2.071401 -0.691136
O 4.340890 -0.125593 0.903857
H 5.300138 -0.111866 0.998448
H 4.125702 -0.869296 0.282800

Na9Cl7(C2HO3)(H2O)+, IXc
E = -5062.275219
C 0.366951 -1.294522 1.717035
C 0.610163 -2.585594 0.887344
O -0.348025 -2.997242 0.189458
O 1.338311 -1.166348 2.722215
O -0.938981 -1.203124 2.260614
O 1.771212 -3.038845 0.938651
Na 3.422441 -2.120293 -0.251487
Na -0.155498 -2.851234 -2.071934
Na -2.478560 -2.199086 0.473713
Na 2.428502 0.953583 2.603388
Cl 4.106556 0.231569 0.695272
Cl 1.821626 -1.102577 -2.206233
Cl -2.262504 -1.252947 -2.154614
Na 3.243830 1.183048 -1.608596
Cl 1.340633 2.843085 -2.035425
Na -0.034376 3.648929 0.027693
Cl -4.199749 -0.453715 1.324355
Na -2.117210 1.115872 1.906284
Na -4.096049 0.484486 -1.105791
Cl 0.251693 2.352732 2.314966
Cl -2.199550 2.247805 -0.638577
Na -0.435392 0.864232 -2.275558
H -0.961681 -1.690459 3.096890
H 0.433434 -0.449957 1.023600
H 1.932855 -1.930504 2.595261

Na9Cl7(C2HO3)(H2O)+, IXd
E = -5062.272998
C 0.934315 -2.710948 -0.953196
O 0.198496 -3.240015 -2.027491
O 2.101860 -3.444590 -0.665790
C 0.003054 -2.571900 0.278632
O -1.225375 -2.513893 0.072071
O 0.590287 -2.455480 1.385491
Na -3.143426 -1.948058 1.051157
Na -0.105839 -1.128762 3.090458
Na 2.821128 -1.762637 1.112068
Na -1.768827 -1.631236 -2.081311
Cl -4.089424 -0.789529 -1.055862
Cl -2.253578 0.188168 2.402386
Na -3.790484 1.377691 0.392547
Cl -2.118396 3.277485 -0.022780
Na -0.216894 2.109358 1.426588
Cl 1.685774 2.923073 -0.565784
Na -0.600505 2.769681 -2.039714
Cl -0.015170 0.293766 -2.868302
Na 2.456771 0.932447 -2.202472
Cl 4.127096 -0.201185 -0.540777
Na 3.528719 1.840631 0.994020
Cl 1.848642 0.548356 2.602955
H 1.857985 -4.345548 -0.409826
H 1.295559 -1.707963 -1.206564
H 0.768937 -3.250114 -2.808445

Na10Cl8(C2HO3)(H2O)+, Xa
E = -5684.965276
C -5.284870 0.631954 0.376052
C -3.986147 0.056954 0.983112
O -2.909872 0.625296 0.627889
O -5.267699 1.363859 -0.584215
O -4.124787 -0.926811 1.720877
Na -2.472640 -2.376010 2.129355
Na -1.110407 1.768668 1.899212
Na -3.118134 1.754918 -1.495391
Na -1.032022 -0.465990 -0.327519
Cl -0.632829 2.503364 -0.707840
Cl -1.324212 -3.275153 -0.111777
Na -0.867020 -2.618192 -2.671110
Na 1.822117 3.323979 0.123063
Cl 3.037373 2.067787 -1.808721
Na 3.826350 -0.315380 -0.979513
Cl -1.838976 -0.150414 -2.947018
Cl -0.245813 -0.845274 2.386650
Na 2.181932 0.273927 3.018414
Cl 3.510381 -1.413237 1.532859
O 5.819616 -1.383761 -0.761964
Cl 1.291168 2.692797 2.616172
Na 1.268648 -2.594445 0.742166
Cl 1.370077 -1.412550 -1.736617
H -6.225149 0.300744 0.843646
Na 0.715216 1.114485 -2.698747
H 6.719202 -1.475251 -1.091123
H 5.797701 -1.757641 0.129557

Na10Cl8(C2HO3)(H2O)+, Xb
E = -5684.960155
C -2.488760 3.764343 -1.035661
O -3.544123 3.519057 -0.503763
C -1.428329 2.669893 -1.253524
O -0.245570 3.044473 -1.194717
O -1.853771 1.488502 -1.380819
Na 1.971579 2.995744 -1.610557
Na -3.737938 1.286580 0.309515
Na 0.012211 0.393359 -0.320406
Na -2.510379 -0.409966 -2.424136
Cl 3.686720 2.446483 0.296840
Na 2.465984 1.977086 2.521876
Cl -1.690314 1.062526 2.011667
Cl -0.665103 -2.139699 -1.509427
Na -0.582280 -1.035672 3.131316
Cl 1.882145 0.487285 -2.444470
Cl -4.331102 -1.078213 -0.690746
Na -2.683393 -2.908541 0.230076
Cl -0.983478 -3.399932 2.151928
Na 1.729260 -2.110357 -2.792951
Cl 3.309527 -2.825623 -0.883717
Na 1.244140 -3.087579 0.718871
Na 3.615302 -0.194818 -0.388003
Cl 1.701750 -0.502381 1.745425
H -2.206364 4.788393 -1.328173
H -0.245976 2.336258 3.086672
O 0.570376 2.700859 3.487705
H 0.297758 3.425026 4.060266

Na10Cl8(C2HO3)(H2O)+, Xc
E = -5684.949191
C 1.089451 -3.222901 -1.490572
C -0.031977 -2.225959 -1.885463
O 0.344854 -1.144812 -2.414006
O 2.349178 -2.663076 -1.787180
O -1.213945 -2.557436 -1.676516
Na 3.791961 -1.848582 -0.133682
Na -2.855756 -2.852249 -0.156014
Na -1.942566 -0.350734 -2.414525
Na 0.965866 0.912516 -1.576012
Na 0.497083 -2.152257 1.935700
Cl -2.084700 -2.518832 2.290096
Cl -3.971412 -0.464160 -0.713326
Cl 3.069507 -1.492361 2.312729
Cl 3.639975 0.688652 -1.083691
Na 4.007809 3.260546 -0.781909
Cl 2.027824 3.530269 0.798725
Cl -1.334696 2.264980 -2.041159
Na 2.471184 1.010456 1.693854
Na -2.684074 0.059052 1.805600
Cl -3.037232 2.699792 1.635583

Na -3.778079 2.207186 -0.785234
Na -0.638374 3.058228 0.654719
Cl 0.001080 0.331734 0.905702
O 1.005341 -3.495664 -0.105533
H 1.202165 -4.426365 0.050400
H 2.152205 -1.869143 -2.321570
H 0.955866 -4.150486 -2.053410

Na10Cl8(C2HO3)(H2O)+, Xd
E = -5684.949942
C -1.388405 3.661145 0.722744
C -1.211218 2.307180 1.456988
O -0.059800 1.958981 1.779801
O -0.169945 4.353142 0.712553
O -2.274990 1.657176 1.613676
Na 1.816021 3.015396 0.849098
Na -3.419796 1.128447 -0.380446
Na -2.605971 -0.336546 2.666665
Na 0.830302 -0.176649 1.795528
Na -0.321843 1.801942 -2.227002
Cl -2.637496 0.737744 -2.833660
Cl -3.591019 -1.431226 0.451798
Cl 2.076059 2.784731 -1.770253
Cl 3.350970 0.795115 1.526170
Na 4.887588 -1.284702 1.227973
Cl 3.966376 -2.143316 -1.001313
Cl -0.628074 -2.007609 2.988394
Na 2.992622 0.374323 -1.242860
Na -2.045027 -1.686267 -1.886528
Cl -0.903963 -4.039765 -1.259078
Na -1.831452 -3.418382 1.098240
Na 1.498219 -3.049267 -1.340579
Cl 0.428984 -0.554728 -0.924172
O -1.817652 3.300399 -0.592759
H -2.214968 4.059163 -1.039668
H -0.301134 5.273938 0.456267
H -2.165573 4.252054 1.213824

Na11Cl9(C2HO3)(H2O)+, XIa
E = -6307.639029
C -4.966725 -1.094685 1.403892
O -4.531894 -1.225763 2.522069
C -4.050964 -0.806730 0.193682
O -2.826082 -0.586599 0.451987
O -4.592573 -0.837950 -0.917596
Na -3.255485 0.002947 -2.549485
Na -1.182751 -1.992427 -0.524462
Na -2.188956 -1.022230 2.745233
Na -1.558565 1.550068 0.272262
Cl 0.579171 -3.454155 -2.039383
Cl -2.964999 2.509191 -1.837477
Na -1.483682 4.572518 -1.725037
Cl 0.329083 3.957228 0.003263
Na 1.504480 -4.035915 0.398965
Cl 3.360443 -2.173964 0.560634
Na 4.821376 0.032051 0.283399
Cl 4.043405 0.678549 -2.126310
Na 2.250202 2.167630 -0.892746
Na 2.413535 -1.429744 -1.954150
Cl -0.633137 1.148205 2.800109
Na 1.106692 3.098461 2.426265
Cl 3.156160 1.639713 1.659587
Cl -0.302420 -2.745337 1.898210
Na 1.404192 -0.528692 1.688302
Cl 0.384029 0.165562 -0.951498
H -6.039963 -1.189517 1.176564
O -2.185912 -2.100240 -2.812169
H -2.790024 -2.827048 -3.010795
H -1.293117 -2.456777 -3.000841

Na11Cl9(C2HO3)(H2O)+, XIb
E = -6307.633726
C -4.963687 -1.180577 -0.770451
C -3.427806 -1.046964 -0.849489
O -2.935148 -1.214196 -1.992063
O -5.553191 -1.434239 0.246979
O -2.784772 -0.814424 0.201048
Na -1.966917 0.540433 -3.062630
Na -2.265832 -1.561504 2.347270
Na -0.829620 -2.115250 -1.107772
Na -1.611654 1.317308 0.302131
Cl -3.101376 2.487642 -1.709432
Na -1.941306 4.666254 -1.082818
Cl -0.024177 3.856334 0.447520
Cl -0.759746 0.662818 2.791655
Na 0.735630 2.828035 2.796574
Cl 2.892075 1.619165 1.923410
Cl 0.268658 0.418377 -1.529019
Cl -0.133001 -2.935150 1.415117
Na 1.926682 -3.992777 0.053575
Cl 3.535582 -1.945745 0.574058
Na 4.818848 0.371554 0.524472
Cl 4.082121 1.168838 -1.839168
Na 2.058458 2.341651 -0.627682
Na 2.681805 -1.094247 -2.012731
Cl 1.198956 -3.275923 -2.342786
Na 1.478330 -0.776397 1.954610
H -5.487528 -1.063056 -1.733306
O -4.408857 -2.043261 2.759786
H -4.924612 -1.832210 1.958938
H -5.016413 -2.424237 3.399977

Na11Cl9(C2HO3)(H2O)+, XIc
E = -6307.626861
C -4.943022 -0.858103 0.385489
O -4.516273 -1.484757 1.614801
C -3.634040 -0.337271 -0.243572
O -2.963078 0.464013 0.439126
O -3.309875 -0.802005 -1.374695
Na -2.144751 0.831869 -2.533461
Na -1.367824 -2.131227 -1.031326
Na -2.467270 -0.685918 2.458393
Na -1.276341 2.028856 0.373403
Cl 0.391995 -3.707606 -2.120917
Cl -2.347281 3.317929 -1.829376
Na -0.605343 5.118103 -1.444928
Cl 0.950467 3.901283 0.236098
Na 0.979586 -4.272956 0.369436
Cl 2.991096 -2.588474 0.629633
Na 4.766417 -0.644961 0.344744
Cl 4.074602 0.135283 -2.051315
Na 2.460068 1.819006 -0.834155
Na 2.252151 -1.802083 -1.981954
Cl -0.488229 1.136800 2.835797
Na 1.535250 2.824270 2.635071
Cl 3.263903 1.107180 1.699562
Cl -0.828762 -2.638158 1.565104
Na 1.210289 -0.780993 1.693004
Cl 0.218330 0.184452 -1.246895
O -5.622330 -1.738646 -0.426106
H -5.020925 -2.010679 -1.137361
H -5.227931 -2.073618 1.896748
H -5.604934 -0.023619 0.632834

Na11Cl9(C2HO3)(H2O)+, XId
E = -6307.608482
C 0.000561 0.431565 -1.388107
O 0.609281 0.150267 -0.324974

C 0.732405 0.301981 -2.746248
O 2.104289 -0.021978 -2.580255
O 0.130011 -0.676166 -3.529548
Na 2.583682 4.469025 -0.816625
Na 2.737214 -2.240501 -2.362929
Na -2.071778 2.701847 -0.088902
Na 1.578216 -1.995449 0.820089
Na -1.435659 -0.708240 0.503567
Na -3.208099 0.152137 -2.331189
Na 1.329835 1.849473 1.084993
Cl -4.462229 1.981150 -0.928197
Na -4.558194 0.286704 1.041025
Cl 3.641875 2.152919 -0.359789
Na 4.360643 0.077457 1.178906
Cl 4.049963 -2.141979 -0.118758
Cl -2.192362 1.054917 2.294269
Cl 0.216801 4.032392 0.064295
Cl 2.117734 0.001331 2.801458
Na -0.372752 -0.502951 3.664638
Cl -0.706244 -2.682688 2.154167
Na -1.806311 -3.832587 -0.041148
Cl 0.300065 -3.113513 -1.497573
Cl -3.621248 -1.838138 -0.482585
O -1.211514 0.779334 -1.428448
H 0.037552 -1.495485 -3.000817
H 2.519150 0.582854 -1.937387
H 0.630158 1.244993 -3.290423

Na3(C2HO3)2+, Ba
E = -1092.135333
C 2.201448 1.421648 -0.552663
C 1.320797 0.952540 0.615597
O 0.388460 1.718093 0.921505
O 2.536904 0.638539 -1.414157
O 1.564546 -0.199154 1.063766
Na -0.087285 -1.227440 2.161590
O -1.748317 -0.599860 0.787143
C -1.357409 -1.027516 -0.328123
C -2.022734 -0.403417 -1.565052
O -2.322834 0.768501 -1.565941
O -0.420935 -1.825992 -0.547341
Na -1.818186 1.743436 0.544952
Na 1.802869 -1.526629 -0.879653
H 2.438677 2.494516 -0.617618
H -2.145260 -1.038108 -2.456510

Na3(C2HO3)2+, Bb
E = -1092.129361
C -3.838350 1.724571 0.000000
C -2.452884 1.044956 0.000000
O -1.470646 1.795813 0.000000
O -4.866700 1.084928 0.000000
O -2.440438 -0.225184 0.000000
Na -4.425879 -1.175159 0.000000
Na 0.000000 -0.000187 -0.000000
H -3.837948 2.824788 0.000000
C 2.452987 -1.045092 -0.000000
O 2.440319 0.225065 -0.000000
O 1.470855 -1.796083 -0.000000
C 3.838541 -1.724467 -0.000000
O 4.866792 -1.084665 -0.000000
H 3.838314 -2.824684 -0.000000
Na 4.425553 1.175446 -0.000000

Na3(C2HO3)2(H2O)+, Ca
E = -1168.601028
C 2.061496 -2.189920 -0.442270
C 0.885302 -1.532676 0.313994
O -0.182299 -2.160917 0.300448
O 3.037069 -1.557568 -0.772830
O 1.125204 -0.404139 0.832835
Na 2.940623 0.664209 -0.077906
Na -2.375571 -1.996840 -0.127807
O -2.396565 0.206242 0.748383
C -2.338157 0.568600 -0.458718
C -1.659634 1.906725 -0.768450
O -0.723350 2.296872 -0.093673
O -2.636794 -0.096222 -1.461977
Na -0.540399 0.760579 1.821807
H -1.975597 2.433844 -1.680307
H 1.941868 -3.253711 -0.698472
O 2.086924 2.735071 -0.345688
H 2.466422 3.595886 -0.553184
H 1.120579 2.845477 -0.372388

Na3(C2HO3)2(H2O)+, Cb
E = -1168.591767
C -2.684736 0.270328 -0.029481
O -2.396119 -0.358557 1.255873
Na -0.782055 -1.962853 1.110042
O 1.423917 -1.540781 0.774765
C 1.975174 -0.465099 0.447336
C 2.247343 0.537164 1.580238
O 2.056547 1.718210 1.404299
C -1.351843 0.139552 -0.798733
O -0.974550 -1.041442 -1.039562
Na 1.021896 -1.603881 -1.841970
O 2.238549 -0.075597 -0.716609
O -0.731062 1.192447 -1.047276
Na 1.308411 2.066306 -0.829930
H -3.481537 -0.298113 -0.513113
H 2.551547 0.133942 2.558951
O -3.093636 1.567803 0.132135
H -2.328545 2.139343 -0.042948
H -3.077029 -0.058806 1.872395

Na3(C2HO3)2(H2O)2+, Da
E = -1245.071586
C 2.096500 -2.618702 0.000000
C 1.189343 -1.366598 0.000000
O -0.030379 -1.581035 0.000000
O 3.301726 -2.540001 0.000000
O 1.775136 -0.243427 0.000000
Na 4.055838 -0.352479 0.000000
Na -2.315136 -1.506035 0.000000
O -2.125318 0.702993 0.000000
C -3.015836 1.606795 0.000000
C -2.471971 3.052187 0.000000
O -1.283816 3.287803 0.000000
O -4.241036 1.474643 0.000000
Na -0.000000 1.274188 0.000000
H -3.224708 3.855550 0.000000
H 1.575952 -3.588766 0.000000
O -4.519551 -1.250033 0.000000
H -5.401604 -1.631107 0.000000
H -4.617474 -0.265618 0.000000
O 6.161254 0.482867 0.000000
H 6.464302 1.398647 0.000000
H 6.963473 -0.053692 0.000000

Na3(C2HO3)2(H2O)2+, Db
E = -1245.066833
C -2.515871 -1.983234 -0.425047
O -3.418978 -1.341422 0.059306
Na -2.837924 0.892159 0.371138
O -1.816682 2.522555 1.534855
C -1.141412 -1.349859 -0.739255
O -1.105505 -0.087313 -0.753644

Na 0.828983 1.094018 -1.128858
O 2.364465 -0.200707 -0.124906
C 2.134973 -0.322693 1.107918
O 2.146179 -1.366558 1.780418
Na 1.886384 -2.511998 -0.234796
O -0.211523 -2.152391 -0.906175
C 1.614234 0.902476 1.864557
O 0.879237 1.705068 1.319416
H 1.840678 0.961001 2.939687
H -2.612113 -3.060948 -0.628448
H -2.100231 3.276293 2.062725
H -0.852111 2.448577 1.647661
O 1.345032 2.538448 -2.834182
H 0.858972 2.767739 -3.634410
H 2.203591 2.969779 -2.919293

Na3(C2HO3)2(H2O)2+, Dc
E = -1245.054738
C -2.804060 0.844780 -0.724853
O -2.217903 1.687443 -0.068115
C -2.600492 -0.644751 -0.435940
O -2.399669 -0.988670 0.761227
Na -1.157450 0.510221 1.817783
O -2.508612 -1.351006 -1.450912
Na -1.146793 -2.758417 -0.177536
O 0.818790 -1.768970 0.226231
C 1.409438 -0.704361 0.474222
O 0.900637 0.362968 0.931987
Na 1.679184 2.183152 -0.209634
O -0.108268 3.542700 -0.426821
C 2.924970 -0.652881 0.170408
O 3.397264 -1.802307 -0.411559
O 3.101393 0.488438 -0.710941
H -3.400069 1.111863 -1.609438
H 3.491363 -0.460750 1.086675
H -0.223953 4.477495 -0.628414
H -0.997705 3.148698 -0.414034
H 2.629982 -2.325279 -0.693960
H 3.927831 0.341986 -1.189384

Na3(C2HO3)2(H2O)2+, Dd
E = -1245.049993
C 2.376275 -0.804611 -0.531494
C 1.854625 0.654647 -0.628614
O 1.319791 0.942057 -1.718531
O 2.205830 -1.307845 0.787891
O 1.963004 1.410148 0.370471
Na 1.200798 0.398291 2.194151
O -0.699657 -0.318100 1.260996
C -1.568022 0.361879 0.638789
C -2.850096 -0.399904 0.235557
O -2.369991 -1.493722 -0.595911
O -1.470207 1.548642 0.278729
O -3.749738 0.382322 -0.442271
Na 0.211668 2.676633 -0.680667
Na -0.255156 -2.168173 -0.052430
H 3.435408 -0.842571 -0.803137
H -3.340507 -0.832498 1.111405
H -3.288317 1.194641 -0.707544
H -3.122785 -1.794540 -1.121333
O 1.610960 -1.614258 -1.402194
H 2.894794 -1.958992 0.967307
H 1.334363 -1.006334 -2.117167
